# Adapting a self-efficacy scale to the task of teaching scientific reasoning: collecting evidence for its psychometric quality using Rasch measurement

**DOI:** 10.3389/fpsyg.2024.1339615

**Published:** 2024-02-07

**Authors:** Virginia Deborah Elaine Welter, Merryn Dawborn-Gundlach, Leroy Großmann, Moritz Krell

**Affiliations:** ^1^Department of Biology Education, IPN – Leibniz Institute for Science and Mathematics Education, Kiel, Germany; ^2^Faculty of Education, The University of Melbourne, Melbourne, VIC, Australia; ^3^Department of Biology Education, Freie Universität Berlin, Berlin, Germany

**Keywords:** self-efficacy, scientific reasoning, rating scale, STEBI, Rasch measurement theory, item response theory, validation

## Abstract

Besides teachers' professional knowledge, their self-efficacy is a crucial aspect in promoting students' scientific reasoning (SR). However, because no measurement instrument has yet been published that specifically refers to self-efficacy beliefs regarding the task of teaching SR, we adapted the Science Teaching Efficacy Belief Instrument (STEBI) accordingly, resulting in the Teaching Scientific Reasoning Efficacy Beliefs Instrument (TSR-EBI). While the conceptual framework of the TSR-EBI is comparable to that of the STEBI in general terms, it goes beyond it in terms of specificity, acknowledging the fact that teaching SR requires very specific knowledge and skills that are not necessarily needed to the same extent for promoting other competencies in science education. To evaluate the TSR-EBI's psychometric quality, we conducted two rounds of validation. Both samples (*N*_1_ = 114; *N*_2_ = 74) consisted of pre-service teachers enrolled in university master's programs in Germany. The collected data were analyzed by applying Rasch analysis and known-group comparisons. In the course of an analysis of the TSR-EBI's internal structure, we found a 3-category scale to be superior to a 5-category structure. The person and item reliability of the scale proved to be satisfactory. Furthermore, during the second round of validation, it became clear that the results previously found for the 3-category scale were generally replicable across a new (but comparable) sample, which clearly supports the TSR-EBI's psychometric quality. Moreover, in terms of test-criterion relationships, the scale was also able to discriminate between groups that are assumed to have different levels of self-efficacy regarding teaching SR. Nonetheless, some findings also suggest that the scale might benefit from having the selection of individual items reconsidered (despite acceptable item fit statistics). On balance, however, we believe that the TSR-EBI has the potential to provide valuable insights in future studies regarding factors that influence teachers' self-efficacy, such as their professional experiences, prior training, or perceived barriers to effective teaching.

## 1 Introduction

Many of the current global challenges (e.g., climate change, environmental pollution, limited resources, epidemics) are closely linked to science (Chowdhury et al., 2020). Engaging in public discussions about such issues and making informed decisions in everyday life requires both knowledge of scientific concepts and a comprehensive understanding of how scientists think and reason (Lederman et al., 2019, 2021). Therefore, scientific reasoning (SR) competencies have become an essential goal of science education. SR competencies are defined as the dispositions to be able to solve a scientific problem in a certain situation by applying a set of scientific skills and knowledge (Krell et al., 2020). It is assumed that SR competencies enable individuals to engage in the process of inquiry, evaluate claims and evidence, draw evidence-based conclusions, and make connections between scientific concepts and real-world phenomena (Osborne, 2013; OECD, 2023). Therefore, science curricula in various countries emphasize the development of SR competencies among students as one factor to foster an informed society (e.g., in Germany: Kultusministerkonferenz, 2004a,b,c). Hence, the development of competencies related to science and scientific reasoning is seen as centrally important in enabling democratic co-determination in science- and technology-based societies (e.g., Lawson, 2004; European Commission, 2015).

However, teaching SR poses several characteristic challenges. First and foremost, SR involves the integration of complex cognitive processes that can be challenging for both teachers and students. Teachers must navigate between supporting students' conceptual understanding, guiding inquiry-based activities, and fostering analytical skills, reflection, and knowledge transfer. In addition, the dynamic nature of scientific inquiry often requires teachers to adapt instructional strategies and engage students in active and collaborative learning experiences. Balancing these different components is an educational challenge, especially when faced with time constraints, limited resources, and standardized curricula that (still) prioritize content coverage over inquiry-based learning (Lederman et al., 2019, 2021).

These challenges, in turn, are directly related to the professional competence of science teachers. To effectively promote students' SR competencies, teachers themselves must be highly competent in this area (Khan and Krell, 2019). On the one hand, such a high level of competence strongly depends on appropriate professional knowledge about SR and adequate instructional approaches (Krell et al., 2023). On the other hand, specific self-efficacy beliefs also play a crucial role in teaching SR. Self-efficacy beliefs are motivational factors that can influence behavior in a given situation (Bong and Skaalvik, 2003). For teachers, self-efficacy beliefs, that is, teachers' expectations regarding their ability to competently manage specific instructional challenges, play a critical role in shaping instructional practices. Studies have shown that teachers who perceive themselves as competent in teaching SR are more likely to use effective instructional strategies, provide appropriate support to students, and persist when facing challenges than teachers with low SR-specific self-efficacy, who may avoid or minimize opportunities for students to engage in SR tasks (Richardson and Liang, 2008; Nie et al., 2013; Mesci et al., 2020).

Therefore, in order to specifically support teachers in promoting SR, it is important to strengthen their professional competence and, in particular, their self-efficacy beliefs. In this context, accurate psychometric measurement is essential to gain valuable insight into the factors that influence teachers' self-efficacy, such as their professional experiences, prior training, and perceived barriers to effective teaching. The results of such assessments are an essential prerequisite for implementing specific training in university teacher education and developing professional development programs that meet teachers' needs. Targeted support for (pre-service) teachers can, in turn, enable them to cultivate SR among their students and help shape a generation of scientifically literate individuals who are prepared to succeed in today's modern world.

However, no measurement instrument has yet been published that specifically refers to self-efficacy beliefs in teaching SR. To address this desideratum, we modified the Science Teaching Efficacy Belief Instrument (STEBI; Riggs and Enochs, 1990) and adapted it to the task of teaching SR. We named the outcome the “Teaching Scientific Reasoning Efficacy Beliefs Instrument” (TSR-EBI). The aims of this study are twofold: Firstly, we provide evidence for the validity of the TSR-EBI to provide a useful measurement for self-efficacy related to teaching SR. Secondly, in reporting the results, we aimed to attach particular importance to a detailed explanation of our approach to rating scale validation because several authors hint to research papers with misapplications of IRT modeling (e.g., Liu, 2020). Hence, the present study will be relevant for scholars interested in teachers' self-efficacy related to teaching SR, but also for those interested in evaluating rating scale instruments in other areas.

### 1.1 Self-efficacy

Convictions about one's self are assumed to help explain and predict individual thinking, experience, and behavior (Bandura, 1994). Self-efficacy beliefs (convictions about being able to act in an intended manner; Bandura, 1997) are related to the more global self-concept (sum of self-defining judgments of who one is; MacKinnon, 2015) and to self-esteem (emotionally shaped self-beliefs; MacKinnon, 2015) in complex ways, which are difficult to differentiate between empirically (Marsh et al., 2019). Furthermore, self-efficacy beliefs show complex intra-relationships. They are assumed to be hierarchically organized (Bong, 1997), with more general self-efficacy beliefs (e.g., about teaching science) comprising more specific ones (e.g., about teaching biology, chemistry, or physics).

The origin of the concept of self-efficacy beliefs is Bandura's (1977, 1986) social-cognitive theory (SCT). Following Bandura (1997), “[p]erceived self-efficacy refers to beliefs in one's capabilities to organize and execute the courses of action required to produce given attainments” (p. 3). In contrast, outcome-expectancies concern predictions about the consequences of certain behavior (Bandura, 1997). Individual self-efficacy beliefs are critical motivational factors that influence behavior in a given situation (Bong and Skaalvik, 2003; Schunk and Meece, 2006). It is assumed that four different sources contribute to their formation and modification (Bandura, 1997; Lazarides and Warner, 2020): (1) previous experience of coping with equivalent situational demands; (2) comparative observation of the successful behavior of others; (3) verbal persuasion (“You can do it”); and (4) physiological and affective conditions (e.g., heart palpitation vs. calmness).

Already from Bandura's (1977, 1997, 2001) considerations, the importance of self-efficacy beliefs for teaching and learning becomes obvious. More specifically, considering the cause-and-effect sequence from teacher education to teacher action to students' learning success (Blömeke et al., 2022), empirical findings point to teachers' self-efficacy as a crucial factor at all three levels (Valentine et al., 2004; Klassen and Usher, 2010; Honicke and Broadbent, 2016; Talsma et al., 2018). For example, several studies have shown that students of teachers with high self-efficacy achieve better learning outcomes (Goddard et al., 2000; Mohamadi and Asadzadeh, 2012; Chambers et al., 2016). High levels of self-efficacy also seem to support teacher wellbeing, confidence, and resilience (Clinton et al., 2018; Ballantyne and Retell, 2020) and, thus, to protect against burnout (Zee and Koomen, 2016). For the domain of science education, positive relationships between high levels of self-efficacy and the use of constructivist, inquiry-based teaching approaches (Richardson and Liang, 2008; Nie et al., 2013), as well as the use of teaching approaches that explicitly target procedural and epistemic scientific knowledge, have been found (Mesci et al., 2020).

However, with respect to teachers' self-efficacy beliefs, the consideration of a certain domain- or task-specificity is self-evident (Bandura, 1994, 1997). For example, a teacher who teaches the subjects of history and mathematics may have higher self-efficacy beliefs in teaching history than in teaching mathematics (possibly because they may have always been more passionate about history). Accordingly, intra-individual differences in self-efficacy beliefs might be found among teachers who teach multiple subjects (Menon and Sadler, 2017; Al Sultan, 2020). In addition, however, inter-individual differences between teachers of different (groups of) subjects may also be expected (Riggs and Enochs, 1990; Welter et al., 2022). In this respect, it is plausible, for example, that teachers who teach comparatively abstract subjects such as chemistry or physics have a different teaching-related self-efficacy than teachers who teach a physical subject such as sports, e.g., due to varying predictability of their teaching and/or of sources of student difficulties (Raudenbush et al., 1992; Ross et al., 1996). Such differences are also reflected in findings on synergy effects among teaching subjects. For example, a study by Welter et al. (2022) showed that pre-service teachers studying both biology and chemistry had both better biology-specific professional knowledge and higher self-efficacy beliefs about experimentation in the classroom compared to those studying only one of these two sciences and a non-science second subject. From this, the authors concluded that an increased semantic relatedness between teaching subjects might have beneficial effects on specific aspects of teachers' professional competence (e.g., via more specific learning opportunities and/or via transfer effects facilitated by discipline resemblance; Welter et al., 2022).

### 1.2 Science teachers' self-efficacy beliefs

There have been several efforts to develop measurement instruments that can assess the specific self-efficacy beliefs associated with science teaching. However, most of these attempts originate from the most popular measurement instrument in this domain, the STEBI (Riggs and Enochs, 1990). This instrument explicitly focuses on science teaching because more global self-assessments (e.g., teaching in general) do not necessarily reflect beliefs about being able to effectively teach science in particular (Al Sultan, 2020). The scale is conceptually aligned with Bandura's (1986) SCT by comprising the two factors of Personal Science Teaching Efficacy (PSTE) and Science Teaching Outcome Expectancy (STOE). The instrument is available in two versions, STEBI-A (Riggs and Enochs, 1990) for use with in-service teachers (practicing teachers who already completed their teacher education program) and STEBI-B (Enochs and Riggs, 1990) for use with pre-service teachers (student teachers in a teacher education program). In both versions, respondents are asked to rate their agreement with each of the items on a 5-point Likert scale (1 = *strongly disagree*; 2 = *disagree*; 3 = *uncertain*; 4 = *agree*; 5 = *strongly agree*). Moreover, both versions include a PSTE and a STOE scale, but the STEBI-B has two fewer items on its PSTE scale and is worded in the future tense. At least two critical aspects should be noted, however, with regard to the STEBI-B version: On the one hand, Bandura (2006) has pointed out that items assessing self-efficacy beliefs should be phrased in the present rather than the future tense as a matter of validity, because many people tend to overestimate their abilities in the future. On the other hand, many researchers consider the STOE subscale (compared to the PSTE subscale) problematic and have therefore removed it from their research designs (e.g., Cannon and Scharmann, 1996; Andersen et al., 2004; McDonnough and Matkins, 2010; Velthuis et al., 2014). However, the comparatively low validity and reliability of the STOE subscale of the STEBI-B version can plausibly be attributed to the fact that pre-service teachers typically do not yet have sufficient practical teaching experience, and thus the necessary conceptualizations of the teaching profession, to adequately respond to the STOE items (Cannon and Scharmann, 1996).

Despite these methodological objections regarding the STEBI-B version, the STEBI has been used many times and in several ways over the past 30 years in research on the personal factors of science teachers' professional competence (Shroyer et al., 2014; Deehan, 2017). For example, Menon and Sadler (2017), as well as Ramey-Gassert et al. (1996), used the instrument to demonstrate the importance of learning opportunities and positive experiences in the context of science for the development of science teachers' self-efficacy beliefs. On the other hand, Mesci et al. (2020), who considered self-efficacy beliefs as a predictor, were able to show that these are a crucial influencing factor for effective teaching on the nature of science and the nature of scientific inquiry. Furthermore, in their recent study on promoting spatial reasoning in STEM subjects, Gagnier et al. (2021) showed that STEBI scores correlate moderately positively with general aspects of teacher self-efficacy (student engagement, instructional strategies, and classroom management) but, in addition, appear to systematically reflect task-specific aspects beyond the general aspects.

Meanwhile, the STEBI's proven usefulness in various research designs has inspired diverse and valuable adaptations and specifications of the instrument to different languages, domains, and tasks (Shroyer et al., 2014). By extending the level of specificity, other domain-specific instruments have been developed, including instruments for use with chemistry teachers (Rubeck and Enochs, 1991), mathematics teachers (Enochs et al., 2000), outdoor educators (Holden et al., 2011), or environmental education teachers (Sia, 1992). Furthermore, the Teaching Science as Inquiry instrument, developed by Smolleck et al. (2006), is one example of a task-specific self-efficacy measure that moves beyond the STEBI's general framework in order to assess science teachers' self-efficacy beliefs in promoting their students' ability to engage in scientific inquiry.

In adapting the STEBI to the specific task of teaching SR, we have also taken such a step. Accordingly, while the conceptual framework of the TSR-EBI is comparable to that of the STEBI in general terms (as evidenced by the wording of the items), it goes beyond Riggs and Enochs' (1990) instrument in terms of specificity, as it is not about teaching science in general, but about teaching SR (in the course of teaching science) in particular. With this higher degree of specificity, we acknowledge the fact that teaching SR requires very specific knowledge (especially own SR competencies) and skills (e.g., effective implementation of specifically effective instructional approaches such as inquiry-based learning) that are not necessarily needed to the same extent for promoting other competencies in science education (e.g., teaching subject matter knowledge about mitosis and meiosis; Khan and Krell, 2019).

In the context of such adaptations, however, particular caution is required. Although, due to the specificity of self-efficacy beliefs, it is reasonable to adapt or specify rating scales that assess self-efficacy in each domain or task (Smith et al., 2003), there is always the danger of developing measures that lose validity. Even if it is possible to find the balance between specificity and predictivity (Bandura, 1994, 1997) and even if the same set of categories and the same labels are used after modification, it seems unreasonable to assume that a rating scale designed to assess self-efficacy in Domain or Task A (e.g., teaching science) will be used in an identical manner by respondents in Domain or Task B (e.g., teaching mathematics). Therefore, appropriate methods should be used to evaluate the psychometric quality of adapted instruments in each individual research setting (American Educational Research Association et al., 2014). In addition to making sure that the measurement instrument accurately measures the intended trait, an important question is that of the specific spacing between a rating scale's categories, and thus whether the number of categories is appropriate. Several researchers have proposed criteria for assessing the quality of rating scale category structures. Such criteria help answer whether a higher category score is related to a higher expression of the measured trait. In other words, applying these criteria can be very useful in identifying categories that provide little or no information (e.g., because they are selected with only low frequency or are redundant to other categories). In such cases, relabeling or collapsing categories may be possible (e.g., Linacre, 2002; Van Dusen and Nissen, 2019). For a comprehensive overview of different types of rating scales, their specific characteristics, and design recommendations, see, for example, Menold and Bogner (2016).

### 1.3 Item response theory and Rasch measurement in test validation

Most testing in psychology and education is based on the classical test theory (CTT), which is also called “true score theory” because it assumes an individual test score to be a directly measurable but biased representation of the underlying latent trait (e.g., intelligence, mathematics skills, self-efficacy). Accordingly, the CTT defines two sources of variance in test scores: the true score variance (due to differences in the latent trait) and the measurement error, which is considered to be unsystematic (DeMars, 2018). Hence, the relationship between the true score and the observed test score can be represented as a linear one. This low statistical complexity is one of the reasons for why the CTT is still widely used today. Item response theory (IRT) models, on the other hand, are based on much more complex assumptions as they describe the probability of correctly answering an item or agreeing with a category as a probability function of the latent trait (person parameter) and specific item characteristics (Cohen et al., 2021).

Both CTT and IRT approaches provide indications of the validity and the reliability of test scores and, to some extent, starting points for test improvement. Regarding the CTT, however, some weaknesses have been discussed concerning the test-dependence of item parameters, the sample-dependence of coefficient measures, and its estimation of measurement error (e.g., Hambleton and Jones, 1993). Many of these aspects are addressed by the IRT, intending to reflect more accurately the relationship between the measurement process and the latent trait being measured. Thus, IRT models offer a powerful methodological framework to evaluate psychometric quality by providing a wide range of fit indices, with some making it possible to evaluate the overall model and others referring to the scale or item level (Hambleton and Jones, 1993; Andrich and Marais, 2019).

Depending on the number of varying item parameters, different hierarchically nested (i.e., increasingly less restrictive) classes of models are distinguished (van der Linden and Hambleton, 1997). Based on the work of Lord (1952), Birnbaum introduced two models in 1968, the 2-parameter logistic (2PL) and 3PL models (Birnbaum, 1968). While the 2PL allows for varying item difficulties and discriminations, the 3PL additionally accounts for guessing. The less common 4PL model (Barton and Lord, 1981) additionally takes slipping effects into account.

Rather independently of Lord's early work, however, the Danish mathematician Georg Rasch presented a model as early as 1960 which, retrospectively, can be understood as a special case of the 2PL IRT models by introducing the restriction of non-varying discrimination indices (i.e., by allowing only varying item difficulties) (Rasch, 1960). Accordingly, the Rasch model is nowadays also referred to as the 1PL IRT model. Originally, the Rasch model was designed for dichotomous items, but meanwhile several extensions for polytomous items are available, e.g., the graded response model (GRM; Samejima, 1969), the rating scale model (RSM; Andrich, 1978), or the partial credit model (PCM; Masters, 1982; Wright and Masters, 1982). Despite its mathematical equivalence to the 1PL IRT models, some practitioners still consider the Rasch measurement theory (RMT) to be a fundamentally independent paradigm (Andrich, 2004). The reason for this is that the IRT and the RMT are based on a different methodological concept: while the IRT follows a model-to-data approach, the RMT follows a data-to-model approach. Consequently, the RMT requires generating new data if they do not fit the specified model, while the IRT instead searches for a model with a better fit. Nonetheless, the statistical procedure is the same for both approaches (van der Linden, 2016), which is why most people, including us, consider models of the Rasch family to belong to the IRT (von Davier, 2016; Cohen et al., 2021). In this study, we used the RMT to evaluate several key validity aspects of the TSR-EBI.

### 1.4 Study aims and research questions

Over the past decades, the IRT has been increasingly used to develop and evaluate test instruments in science education research. However, Liu (2020) pointed out that with this increased use, the number of research papers in which casual or even misapplications of IRT modeling can be found has inevitably increased as well. In this context, and specifically regarding Rasch modeling, Planinic et al. (2019), for example, pointed out that “[…] it is likely that the Rasch approach is not generally well understood […]” (p. 1). Similarly, Oon and Fan (2017) stated that “[…] although Rasch model has been receiving some more attention in science education, many researchers in this field are probably still unaware of what Rasch analysis can offer, and of how Rasch analysis can help in improving the psychometric quality of assessment in science education research” (p. 2). Therefore, we had two aims in this study: first, we aimed to collect evidence of the validity of the TSR-EBI to provide a useful measurement instrument that may be fruitful for future research and/or the development of training and professional development programs to improve self-efficacy related to teaching SR. Second, in reporting our results, we aimed to attach particular importance to a detailed explanation of our methodological approach for those readers who are not yet familiar with Rasch model applications in rating scale validations. With this in mind, we aimed to answer a total of seven research questions (RQ), which are presented below. RQ 1 through RQ 6 were exploratory in nature, while RQ 7 was based on a specific hypothesis.

- Does the empirical TSR-EBI data matrix meet the general assumptions of Rasch measurement?
- To which of two models considered for describing rating scale data (see Section 2.2) does the empirical TSR-EBI data matrix fit better: the RSM (Andrich, 1978) or the PCM (Masters, 1982; Wright and Masters, 1982)?
- Is the number of rating scale categories selected in the TSR-EBI appropriate or can it be refined in terms of measurement quality?
- Is the TSR-EBI's item selection appropriate or should individual items be removed from (or added to) the scale due to insufficient measurement quality?
- Is the TSR-EBI's reliability supported?
- Are the TSR-EBI's results replicable in a different (but comparable) sample?
- How does the TSR-EBI perform in a known-groups comparison? Do the test scores discriminate between groups that are assumed to have different levels of self-efficacy in teaching SR? In this respect, we hypothesized that pre-service teachers studying two science subjects would achieve higher TSR-EBI scores than those who took only one science and a non-science second subject (see Section 2.2). The underlying assumption for this hypothesis was that pre-service teachers who study two science subjects may generally benefit from more learning opportunities both in SR itself and in teaching it and, therefore, could be expected to show higher self-efficacy beliefs (Heitzmann, 2002; Welter et al., 2022).

## 2 Materials and methods

To develop the TSR-EBI, STEBI items (Riggs and Enochs, 1990) were translated from English into German and adapted to the task of teaching SR (the final items can be found in the Supplementary material). The test development consisted of several rounds of discussion and revision. The main topics of these sessions were content specification, wording, scale format, and administration. According to American Educational Research Association et al. (2014), such a discursive analysis of the relationship between test content and the construct that is to be measured represents an essential basis for an instrument's validity. Specific questions raised during the test development concerned, for example, which STEBI (Riggs and Enochs, 1990) version should be used or whether only one or both subscales (PTSE and STOE) should be adapted. Although the TSR-EBI focuses on the population of pre-service teachers, we referred to the STEBI-A version (Riggs and Enochs, 1990) as Bandura (2006) pointed out that the future-tense wording of the self-efficacy items (as used in the STEBI-B version of Enochs and Riggs, 1990) is not recommended. Moreover, we considered only the PSTE scale (Riggs and Enochs, 1990) in our adaptation to avoid problems associated with the STOE scale regarding its actual predictivity for self-efficacy due to pre-service teachers' limited practical teaching experience (Deehan, 2017; see Section 1.2). Moreover, due to this lack of experience, it was also important to include a brief written definition of what teaching SR entails (“promoting students' knowledge about methods of acquiring scientific knowledge”) in the test instructions to increase the likelihood that all participants would be thinking of the same construct when answering the items. In contrast to the STEBI-A (Riggs and Enochs, 1990), which asks about the extent to which participants agree with each item, in the TSR-EBI, we asked our participants to state for each item how much it applies to them personally. Initially, the scale format was specified as a 5-point rating scale (1 = *not at all*; 2 = *slightly*; 3 = *moderately*; 4 = *very*; 5 = *totally*). However, as our statistical analyses indicated that the category structure needed to be optimized (see Section 3), it was revised, resulting in a 3-point rating scale [1 = *(rather) not*; 2 = *moderately*; 3 = *(rather) strongly*; see Section 2.2].

### 2.1 Samples and procedure

Because the above-described process of optimizing a rating scale might be sample-dependent (Smith et al., 2003), it was necessary to retest the revised scale with a new sample from the same population. Therefore, the remaining article describes two related data collections: a first round of validation and a cross-validation.

Both samples consisted of pre-service teachers who were completing their university master's degree in Germany. German teacher education consists of two consecutive phases: the first phase is a 5-year academic part, culminating in a master's degree. This first phase covers learning opportunities in at least two teaching subjects as well as in subject-specific education, general education, and psychology. Successful completion of this phase grants admission to the second phase, which lasts about 18–24 months and primarily comprises practical training in school to gain teaching experience (Terhart, 2019).

The participants in our first round of validation were pre-service teachers studying at one public university in the northeast of Germany (*N* = 114). All of them intended to acquire degrees in two teaching subjects, one of which was biology. Regarding the second subject, there were 17 (14.9%) participants who studied another natural science (chemistry or physics) next to biology, 10 (8.8%) whose second subject was mathematics, and 87 (76.3%) who studied biology and any subject other than chemistry, physics, or mathematics (mainly from the humanities, e.g., languages, geography, social sciences).

The participants in our cross-validation were pre-service teachers studying at one public university in the west of Germany (*N* = 74). Just like in the first round of validation, all participants intended to acquire degrees in two teaching subjects, one of which was biology. There were nine (8.1%) participants whose second subject was another natural science (chemistry or physics), 6 (17.6%) whose second subject was mathematics, and 55 (74.3%) whose second subject was any one other than chemistry, physics, or mathematics (again mainly from the humanities).

Participants in both samples were recruited by the authors and their colleagues during university lectures in biology education. Participation was voluntary and completely anonymous. Except for the pre-service teachers' majors, no personal information was collected, ruling out any possibility of identifying individual respondents. Ethical approval was obtained from the IPN's local ethics committee (ID: 2021_KR43). Data collection took place online at a time most convenient for participants. All pre-service teachers interested in participating in the study were given a link to the questionnaire. The time needed to complete the questionnaire was ~3–5 min in both rounds of data collection, but there was no time limit. In the first round of validation, there were missing values in only one data set, so a total of 113 complete data sets were available. In the cross-validation, there were no missing data. For the analysis of data from polytomously scored items, Linacre (1994) suggests a minimum sample size of 50 to be appropriate “for most purposes.”

### 2.2 Statistical analyses

Two established models for polytomously scored items (e.g., those of a rating scale) are the RSM (Andrich, 1978) and the PCM (Masters, 1982; Wright and Masters, 1982; see Section 1.3). Both models belong to the Rasch family and are used to specify category response functions. Furthermore, in both models, the rating scale thresholds are usually specified as Andrich thresholds (i.e., points where there is a 50.0% chance of scoring in one of two adjacent categories), not as Thurstone thresholds which refer to the cumulative probability of being rated below or above a particular category (Masters, 1992).

According to the RSM, the probability that person *i* with ability θ_*i*_ scores in one of the response categories *x*_*ij*_ ∈ {0, 1, …, *p*} of item *j* with item location parameter β_*j*_ and a set of threshold parameters τ is estimated as follows (Komboz et al., 2018):


$$\begin{array}{l} P\left(X_{ij}=\;x_{ij}|\theta_{i},\beta_{j},\tau \right)=\frac{\exp\sum_{k=0}^{x_{ij}}\left(\theta_{i}-\left(\beta_{j}+\tau_{k}\right)\right)}{\sum_{\ell =0}^{p}\exp\sum_{k=0}^{\ell }\left(\theta_{i}-\left(\beta_{j}+\tau_{k}\right)\right)},\sum_{k=0}^{0}\equiv \;0 \end{array}$$

**Figure 1:**
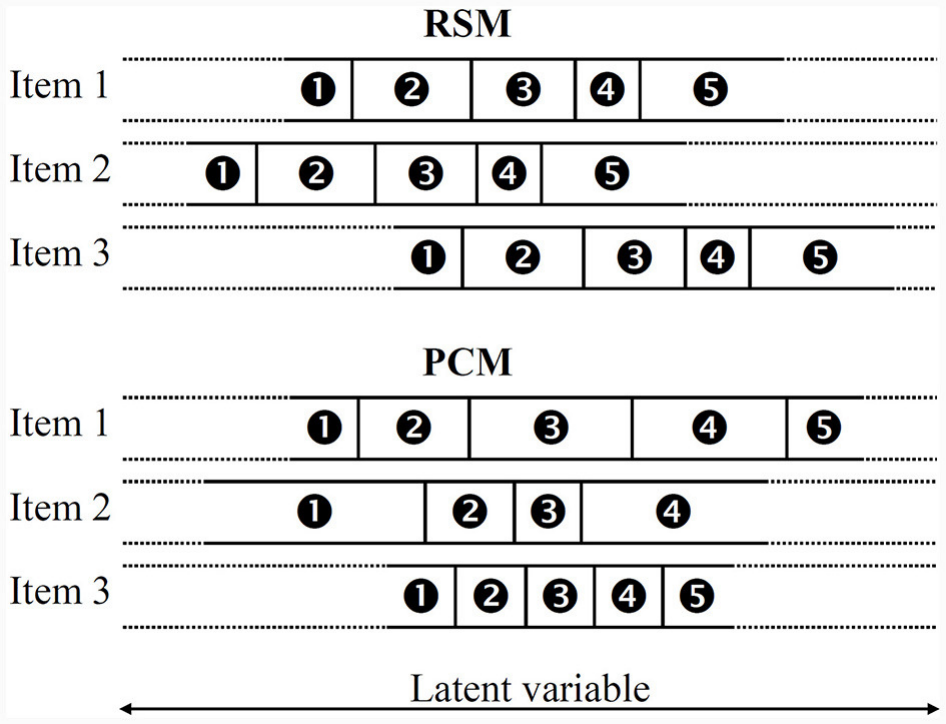
Three prototypical items that fit either the RSM **(top)** or the PCM (**bottom**; adapted from Linacre, 2002).

From the above, it can be seen that the RSM describes items by two different parameters: while β_*j*_ relates to the overall location of item *j* on the latent scale, the threshold parameters τ = (τ_1_, …, τ_*p*_) indicate the distance between β_*j*_ and the rating scale thresholds (points where there is a 50.0% chance of scoring in one of two adjacent categories). In this regard, the RSM specifies these threshold parameters τ to be constant across all items, that is, it assumes all items to share the same set of categories and each individual category to have an equal range across all items. In contrast, the PCM is less restrictive in that it allows both a variable number of categories and variable ranges of each individual category per item (see Figure 1). Thus, PCMs describe items by only one set of threshold parameters δ_*j*_ = (δ_*j*1_, …, δ_*j*_*p*__*j*__), which correspond to those points at which the probability of scoring in category *k* is equal to that of scoring in category *k* − 1 (Komboz et al., 2018):


$$\begin{array}{l} P\left(X_{ij}=\;x_{ij}|\theta_{i},\delta_{j}\right)=\frac{\exp\sum_{k=0}^{x_{ij}}\left(\theta_{i}-\delta_{jk}\right)}{\sum_{\ell =0}^{p_{j}}\exp\sum_{k=0}^{\ell }\left(\theta_{i}-\delta_{jk}\right)} \end{array}$$

As part of our analyses, we considered both models and checked which one our data fit better. All statistical analyses were performed using WINSTEPS (Version 5.2.3.0) and SPSS (Version 28). Before conducting our analyses, we reverse coded all negatively phrased items (Items 2, 4, 5, 7, 9, 10, 11, and 13; see section Supplementary Information on the Wording of the TSR-EBI Items in Supplementary material). To estimate the respective model parameters, we used conditional maximum likelihood estimation (CMLE). In contrast to joint maximum likelihood estimation (JMLE), which estimates item and person parameters simultaneously, CMLE considers individual sum scores to be sufficient statistics regarding the person parameter θ_*i*_, allowing the item parameters to be estimated first and then used to estimate the person parameters in a second step (Haberman, 2004).

#### 2.2.1 Assumptions of Rasch measurement (RQ 1)

In a first step, for each of our two models, we checked whether there was a general fit of our data to the three basic assumptions of any Rasch measurement: unidimensionality, local independence, and specific objectivity (Bond et al., 2020).

Unidimensionality means that all person and item parameters of the model refer to only one common construct, that is, the test does not measure any additional latent trait (Bond et al., 2020). Accordingly, the residuals that remain after the extraction of the Rasch measurement should be randomly distributed, that is, they do not represent any meaningful subdimensions. This assumption was tested by conducting a principal component analysis (PCA) of the residuals, which are represented as contrasts (a kind of component but built from residual variance). A common evaluation criterion is that the contrasts should not explain more than 15.0% of the total variance (Fisher, 2007) and should not have eigenvalues that are larger than expectable by chance (≈2.00; Raîche, 2005; Linacre, 2022). In addition, the variance explained by the residual contrasts should be evaluated in relation to the variance that is attributable to the item difficulty (Linacre, 2022).

Local independence means that the response to a specific item is not dependent on the responses to previous items (Bond et al., 2020). Accordingly, after partialing the latent variable out, there should no longer be any meaningful positive correlations between the manifest variables. Therefore, we checked the correlation matrix of the standardized residuals of all 156 pairs of variable combinations for such correlations. Regarding their level, Linacre (2022) states that they “need to be around 0.7 before we are really concerned about dependency” (p. 441).

Specific objectivity means that comparisons of the abilities of two respondents are item-independent (each task is equally suitable) and, vice versa, that item comparisons are independent of person ability (Bond et al., 2020). Accordingly, all items should have the same discrimination. We tested this assumption using van den Wollenberg's (1982) *Q*_1_ statistic. If the corresponding significance test does not reject the null hypothesis, all item characteristic curves have the same slope, that is, they are parallel to each other.

#### 2.2.2 Model fit (RQ 2)

After having ensured that these assumptions of Rasch measurement were met, we used a global model test to answer the question of whether the two models in question each were generally able to predict the empirical data. One test that is suitable for this is, for example, Pearson's (1900) chi-squared test, which compares the frequencies of response patterns predicted by the model with those observed empirically. If there are significant deviations across all patterns, the test rejects the null hypothesis that the model fits the data well.

Once we had checked whether each of the two competing models was able to explain the empirical data matrix, we used comparative measures to evaluate which one should be given preference. Akaike's (1974) information criterion (AIC) considers the likelihood of the data (log-likelihood estimate) and the complexity of the model via the number of parameters considered. In contrast to the AIC, the Bayesian information criterion (BIC; Schwarz, 1978) gives more weight to the complexity of the model, as the number of parameters is weighted by the logarithmic sample size. This acknowledges the fact that one can achieve a better model fit by specifying additional parameters as the sample size increases. For both the AIC and the BIC, the better model is determined by the smaller value. Finally, the root mean square residual (RMSR) considers the sum of squared deviations between the empirical values and those predicted by the model in question. If the observed RMSR value is smaller than the expected one, this indicates better model fit (Linacre, 2022). If a comparison of these measures reveals only minor differences between models, the scope, usefulness, and other practical characteristics of a model can also be taken into consideration when making a decision (Cohen et al., 2021).

#### 2.2.3 Category effectiveness (RQ 3)

After we had selected the model of better fit, we checked this model to decide whether the number of rating scale categories selected was appropriate. Specifically, we evaluated (1) the observation frequency and distribution per category, (2) the category-related coherence indices, (3) the categories' degree of alignment with average measures and point-biserial correlations (each response category's correlation with the overall score), (4) the advance patterns of threshold parameters, and (5) the category-related INFIT and OUTFIT mean-squares (MNSQ). Because the PCM (Masters, 1982; Wright and Masters, 1982) estimates categories separately for each item, it was necessary to consider the abovementioned parameters for each individual item, whereas, for the RSM (Andrich, 1978), only the overall scale was considered due to the assumption of item-invariant threshold parameters.

Since the estimation of threshold parameters is based on the log-ratio of the frequency of the adjacent categories, it becomes more imprecise and unstable as this frequency decreases. Therefore, according to Linacre (2002), at least 10 observations of each category are required. Furthermore, these observations should form a smooth and regular distribution with distinct peaks for each category in a category-probability plot. Such a plot can provide information about the categories' coherence, which strongly depends on how much the category-probability curves overlap. Coherence refers to the questions of how well an estimated measurement can be predicted from an observed category (C→M) and, vice versa, how well an observed category can be predicted from an estimated measurement (M→C). According to Linacre (2002), values ≥40.0% are acceptable. In addition, we had to make sure that increasing category scores were actually associated with higher levels of self-efficacy in teaching SR. For this purpose, we checked whether the average measures (person ability) and the point-biserial correlations (noticeably) increased with increasing category scores. Moreover, for the highest category score, the point-biserial correlation should be positive (Wu and Adams, 2007). Similarly, the threshold parameters should increase monotonically, with steps of at least 1.00 logit (for a rating scale with five categories) or 1.40 logits (for a rating scale with three categories), but <5.00 logits (Linacre, 2002). Finally, we evaluated the category-related INFIT and OUTFIT MNSQ values,^1^ which are based on the sums of the squared residuals associated with the responses in each category. While OUTFIT is an unweighted fit statistic that is sensitive to outliers (extreme deviations from model expectations), INFIT is a variance-weighted fit statistic that is inlier-sensitive due to a smaller variance in extreme observations (Wright and Masters, 1982; Wright and Linacre, 1994). Both INFIT and OUTFIT have an expected value of 1.00, with smaller values indicating overfit (the model predicts the data too well) and larger values indicating underfit (unmodeled noise in the data). Accordingly, high MNSQ values affect validity much more seriously than low ones (Linacre, 2022). Ideally, MNSQ values should range between 0.50 and 1.50. Values ≥1.50 require closer investigation, while values ≥2.00 clearly indicate ineffective measurement (Wright and Linacre, 1994).

#### 2.2.4 Item fit (RQ 4)

To check whether the item selection was appropriate, we first evaluated the item-related INFIT and OUTFIT MNSQ values, which are to be interpreted similarly to their category-related counterparts (Wright and Linacre, 1994). Furthermore, we once again looked at point-biserial correlations. At the item level, they should always be positive in the case of positive items (this was the case here after recoding), as this means that an item score is in line with the orientation of the latent variable (Linacre, 2002). In addition, we evaluated the items' estimated discrimination to assess how accurately the TSR-EBI provides information about the respondents' relative position on the latent variable self-efficacy in teaching SR. If the model is valid, the discrimination has an expected value of 1.00. A value >1.00 means that an item with a given difficulty discriminates better than expected; a positive value <1.00 means that it discriminates worse than expected (Lopez, 1998). Negative discrimination is usually accompanied by negative point-biserial correlations and indicates a contradiction between the orientation of the item and the latent variable (Linacre, 2022). Finally, we had a closer look at the Wright map to evaluate the items' locations in relation to our participants' trait levels. Both parameters are represented along a vertical axis: the pre-service teachers' self-efficacy scores are on the left (from *less* at the bottom to *more* at the top) and the items are on the right (from *easier to agree with* at the bottom to *more difficult to agree with* at the top). The degree of alignment between both is a crucial quality measure regarding the construct validity of an instrument (Wright and Masters, 1982). If items cover only a small part of the trait variation, if there are larger gaps between them, or if several items are located in one position instead of being “spread” across the latent variable, this indicates a need for optimization (Bond et al., 2020).

#### 2.2.5 Reliability (RQ 5)

After we had completed the item analysis, we checked whether the reliability of the TSR-EBI was supported by considering both person and item reliability. While the person reliability, which relates to the person-score-order reproducibility, is comparable to Cronbach's α in the CTT, the item reliability has no such CTT counterpart. It corresponds to the item-value-order reproducibility, that is, it shows whether the item difficulty hierarchy can be stably reproduced for a given sample size (Boone et al., 2014). Low item reliability (<0.90) usually indicates that the sample size is too small to precisely locate the items on the latent variable. On the other hand, low person reliability indicates that either the number of items or the range of the respondents' ability is too small. To increase the likelihood that a test will discriminate the sample efficiently according to the respondents' ability, person reliability values of ≥0.80 are desirable for rating scales (Cohen et al., 2021).

#### 2.2.6 Cross-validation (RQ 6)

After having checked all these quality criteria, we had to make sure that the results we had found could also be replicated independently among another (but comparable) sample. For this purpose, we analyzed the data of our cross-validation to compare them with those obtained from the first round of validation regarding RQ 1 to RQ 5. More precisely, we checked whether there were significant deviations in the pattern of results between the first round of validation and the cross-validation and, thus, whether there were any indications of a lack of the TSR-EBI's psychometric quality.

#### 2.2.7 Relations to other variables (RQ 7)

Finally, to collect validity evidence from the relations to other variables, we checked whether the TSR-EBI test scores sufficiently discriminated between groups that are assumed to have different levels of the measured construct (American Educational Research Association et al., 2014; Cohen et al., 2021). For this purpose, we first combined the data from our first round of validation with those from our cross-validation sample into one data matrix including 188 respondents. We considered this procedure to be acceptable due to the comparability of both samples in terms of semester of study. In a next step, we classified the pre-service teachers according to the teaching subjects they majored in. All of them selected two teaching subjects, one of which was biology. The grouping was carried out depending on whether they combined (1) biology with chemistry or physics (B&C|P group); (2) biology with mathematics (B&M group); or (3) biology with any subject other than chemistry, physics, or mathematics (B&¬[C|P|M] group). Establishing the B&C|P group was based both on previous empirical findings (see Section 1.4) and on the fact that in the German educational standards, the promotion of SR competencies is an explicit learning goal only in the three subjects of biology, chemistry, and physics (Kultusministerkonferenz, 2004a,b,c). However, the B&M group was considered separately due to the unique position of mathematics between the natural sciences and the humanities (e.g., Mager and Hein, 2019). Overall, the frequency distribution was as follows: while both the B&C|P and the B&M group each comprised 23 respondents, the B&¬[C|P|M] group comprised 142 respondents. Due to this imbalanced distribution, we then extracted 10 random samples from the B&¬[C|P|M] group, each comprising 23 respondents. For each of these 10 random samples, we ran an ANOVA to examine the variation in the self-efficacy beliefs of the three groups considered. The dependent variable was operationalized by obtaining weighted likelihood estimates (WLE), which had been calculated during the previously conducted IRT analyses. The results of the 10 ANOVAs were then averaged to generate an overall result that was as informative as possible.

## 3 Results

For the sake of comprehensibility, the following sections are not aligned with the order of the research questions; instead, they are aligned with the chronology of the analyses. Accordingly, we first report on the initial TSR-EBI version; next, on its revision based on these first results; after that, on the cross-validation of the revised version, and, finally, on the merging of the data sets from the first round of validation and the cross-validation in order to answer the question about the TSR-EBI's relations to other variables.

### 3.1 Initial 5-category version of the TSR-EBI

For the initial 5-category version of the TSR-EBI, we only answered RQ 1 to RQ 3 before it became clear that the category structure needed to be revised. Accordingly, an analysis of the item structure (RQ 4) or the reliability (RQ 5) was not conducted for this version.

#### 3.1.1 Assumptions of Rasch measurement (RQ 1)

For the RSM, the total variance explained by the model was 40.6% (18.0% attributable to persons; 22.6% attributable to item difficulties) and, thus, was somewhat lower than that explained by the PCM (43.7%; 19.3% attributable to persons; 24.4% attributable to item difficulties). However, both values were close to the Rasch model predictions of 40.4% (RSM) and 44.3% (PCM), respectively, suggesting that the empirical data matrix fit both models satisfactorily. Regarding the eigenvalue of the first residual variance contrast, both models slightly exceeded the cut-off value of 2.00, with values of 2.12 (RSM) and 2.06 (PCM). At the same time, however, the first contrast explained only 9.7% (RSM) and 8.9% (PCM) of the total variance, which is well below the threshold of 15.0% in both cases. Moreover, for both models, the variance attributable to the item difficulties was more than twice as large. Accordingly, we did not assume multidimensionality for either the RSM or the PCM. With regard to the correlations between the standardized residuals, for both models, the mean was *r* = −0.08 and was thus close to a null correlation. The highest positive correlation was between Item 6 and Item 8 for both models and reached levels of *r* = 0.33 (RSM) and *r* = 0.34 (PCM). Thus, we were also able to assume local independence for both models. Additionally, van-den-Wollenberg's *Q*_1_ statistic turned out to be nonsignificant for both the RSM (*Q*_1_ = 216.24, *p* = 0.11) and the PCM (*Q*_1_ = 193.88, *p* = 0.45), suggesting that specific objectivity could also be assumed. Consequently, both the RSM and the PCM met all three basic assumptions of Rasch measurement.

#### 3.1.2 Model fit (RQ 2)

Table 1 shows the results of the model comparison that was made to evaluate whether our data fitted the PCM or the RSM better. In both cases, the standardized residuals were close to their expected values and Pearson's chi-squared test turned out to be nonsignificant, suggesting an adequate fit of the data to each of the two models. In terms of information criteria, the AIC favored the PCM, whereas the BIC favored the RSM. The RMSRs in both cases were very similar and close to those predicted by each model, indicating that the intended construct is measured without significant interference, regardless of which of the two models is referred to. Overall, against the background of two almost equally fitting models, however, the BIC decision is most likely to be agreed with, as it considers a model's parsimony. The principle of parsimony states that a simpler model with fewer parameters is preferable to a more complex model with more parameters, provided the models fit the data comparably well (Vandekerckhove et al., 2015).

**Table 1:** Fit statistics of the 5-category PCM and RSM.

**Rasch model**	**Standardized residuals**		**Pearson** χ^**2**^ **test**		**AIC**	**BIC**	**RMSR**	
	***M***	***SD***	χ^2^	***p***			**obs**.	**exp**.
PCM (48 estimated parameters)	−0.01	1.01	1,495.06	0.09	3,073.88	3,328.01	0.6996	0.6964
RSM (15 estimated parameters)	0.00	1.00	1,483.65	0.31	3,155.59	3,235.00	0.7070	0.7092

PCM, partial credit model; RSM, rating scale model; AIC, Akaike information criterion; BIC, Bayesian information criterion; RMSR, root mean square residual; obs., observed; exp., expected.

#### 3.1.3 Category effectiveness (RQ 3)

**Figure 2:**
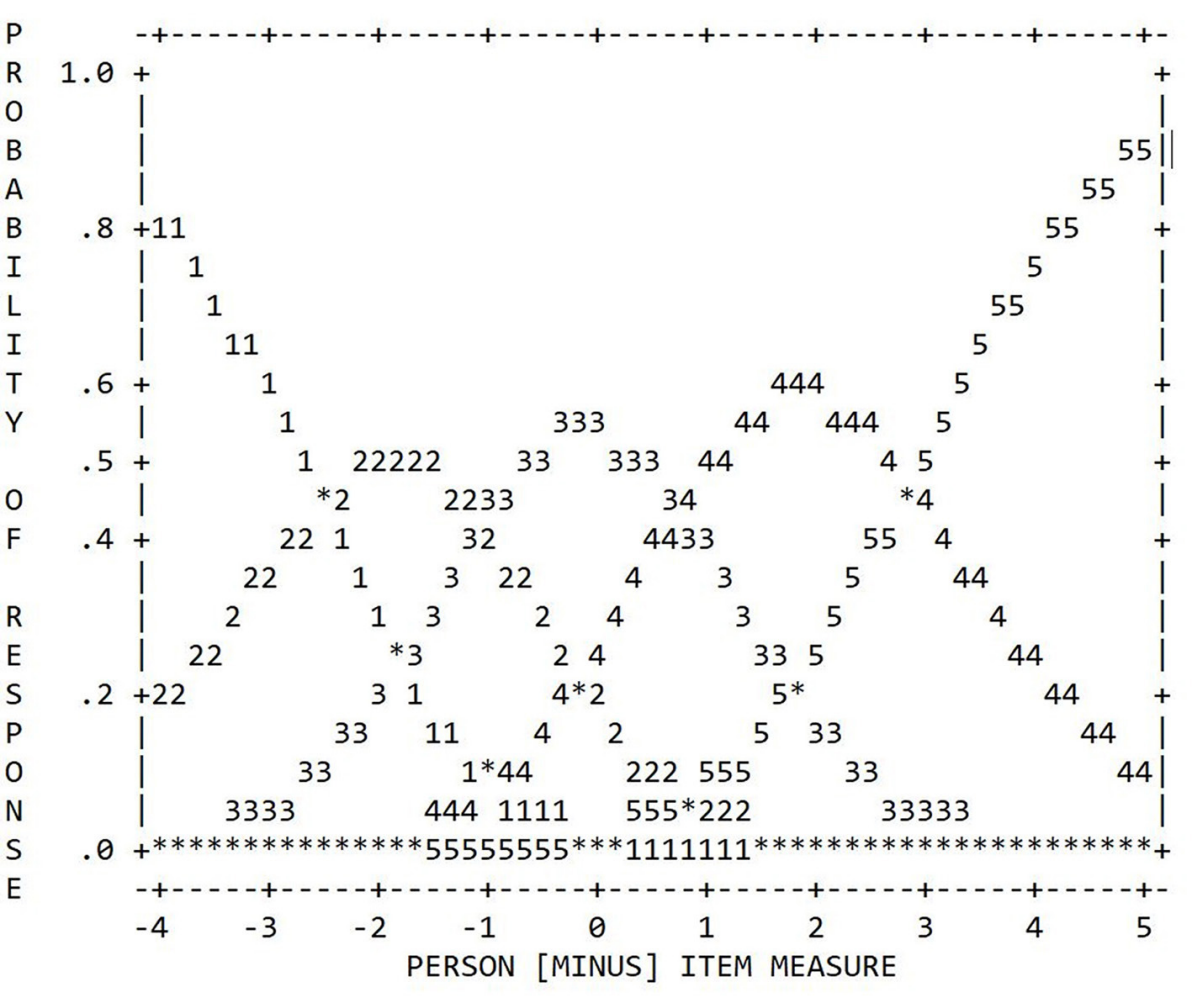
Category probability curves of the 5-category TSR-EBI (RSM).

After having decided that our data better fit the RSM, we proceeded to the category statistics. Our results showed a sufficient number of 10 observations per category (see Table 2) and a regular distribution pattern with distinct peaks for each category in the category-probability plot (see Figure 2). However, Category 1 was used much less frequently than all other categories, while Categories 2 and 5 were used more frequently but still considerably less frequently than Categories 3 and 4. This was also reflected in the categories' coherence. For Category 1, both coherence indices were below the 40.0% level, while Categories 2 and 5 showed satisfactory M→C percentages but unsatisfactory C→M percentages. Furthermore, we found a monotonic increase only for the average measurements and the step difficulties, whereas the point-biserial correlations were disordered for Categories 1 and 2 and had hardly distinguishable levels for Categories 4 and 5. Finally, regarding the INFIT and OUTFIT MNSQ values associated with the responses in each category, all values were close to the expected values of 1.00 except in the case of Category 1. However, even for Category 1, the INFIT and OUTFIT MNSQ values did not exceed values of 2.00.

**Table 2:** Category statistics of the 5-category TSR-EBI.

**Rating scale category**	**Observed count**	**Average measure**	**MNSQ**		**Step difficulty**	**Coherence**		***r*_pbis_**
			**INFIT**	**OUTFIT**		**M**→**C**	**C**→**M**	
1	34	−0.91	1.44	1.59	n.a.	25.0%	3.0%	−0.19
2	181	−0.46	0.92	0.93	−2.30	51.0%	23.0%	−0.27
3	541	0.37	0.95	0.96	−1.00	51.0%	66.0%	−0.16
4	559	1.19	0.92	0.93	0.68	55.0%	65.0%	0.25
5	157	2.01	1.00	1.00	2.62	67.0%	14.0%	0.27

MNSQ, mean square; M→C, measure implies category; C→M, category implies measure; r_pbis_, point-biserial correlation. The underlying model is the rating scale model (RSM). All model parameters were estimated using conditional maximum-likelihood estimation (CMLE).

**Figure 3:**
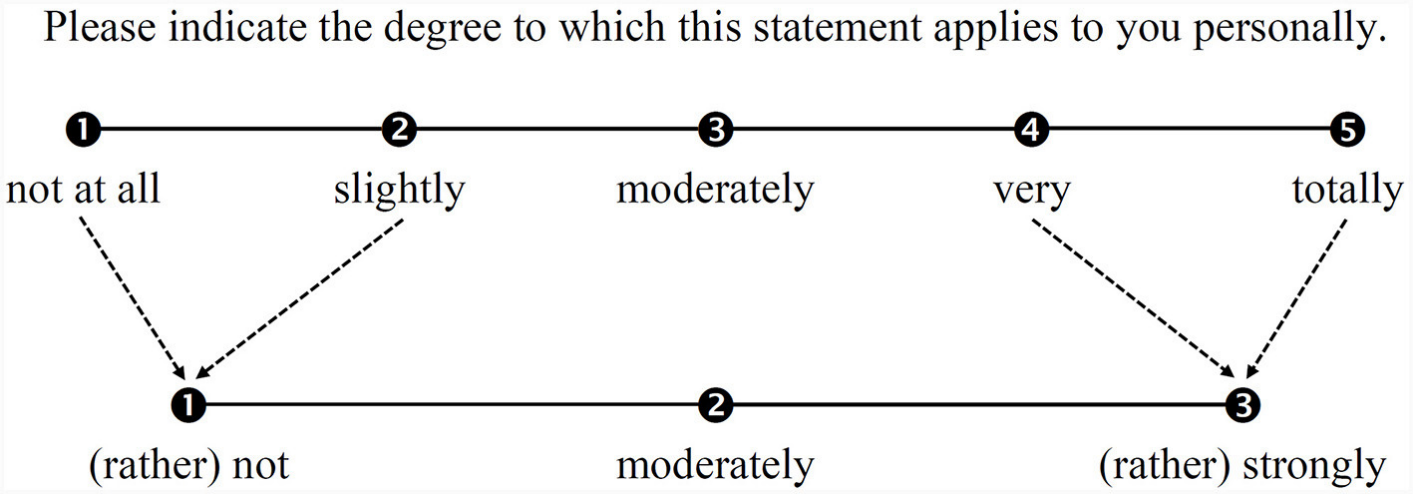
Scheme of collapsing categories of the TSR-EBI's rating scale.

Therefore, overall, our findings for the initial 5-category structure were rather heterogeneous, revealing at least some potential for optimization, above all with respect to Category 1, but also with respect to Categories 2 and 5. Although both the average measures and the step difficulties increased monotonically (the latter even with a satisfactory advance of >1.00 logit), the point-biserial correlations in particular indicated that the exterior categories provided comparatively little or little precise information about the trait of interest. The latter assumption was also supported by the coherence indices. We therefore tried to improve the scale's measurement quality by collapsing Categories 1 and 2 and also Categories 4 and 5 into one category each, resulting in an overall 3-category scale (see Figure 3).

### 3.2 Revised 3-category version of the TSR-EBI

To check whether this remodeling of the category structure actually improved the measurement quality, we first had to re-evaluate the model fit (RQ 1 and RQ 2). Afterward, we re-examined the category effectiveness (RQ 3) before we evaluated the item selection (RQ 4) and the reliability coefficients (RQ 5).

#### 3.2.1 Assumptions of Rasch measurement (RQ 1)

For both the RSM and the PCM, the total variance explained by the model was somewhat below the values found for the 5-category structure. For the RSM, it was 1.1% less, resulting in 39.5% (20.0% attributable to persons; 19.5% attributable to item difficulties); for the PCM, it was 4.2% less, resulting in 40.1% (20.2% attributable to persons; 19.9% attributable to item difficulties). However, both values were again close to the Rasch model predictions of 39.1% (RSM) and 39.6% (PCM), respectively. Regarding the eigenvalue of the first residual variance contrast, this time, both models were below the threshold of 2.00 with values of 1.93 (RSM) and 1.92 (PCM). In terms of the total variance, 9.1% (RSM) and 8.8% (PCM), respectively, were explained by this first residual variance contrast. Accordingly, the assumption of unidimensionality was clearly met for both models. With regard to the correlations between the standardized residuals, for both models, the mean was again *r* = −0.08. The highest positive correlation was between Item 8 and Item 12 for both models and reached somewhat lower levels compared to the 5-category structure: *r* = 0.29 (RSM) and *r* = 0.27 (PCM). Thus, we were also able to assume local independence for both models. Additionally, van-den-Wollenberg's *Q*_1_ statistic again turned out to be nonsignificant for both the RSM (*Q*_1_ = 182.07, *p* = 0.44) and the PCM (*Q*_1_ = 154.81, *p* = 0.76), suggesting that specific objectivity could also be assumed. Consequently, again, both the RSM and the PCM met all three basic assumptions of Rasch measurement.

#### 3.2.2 Model fit (RQ 2)

Table 3 shows the results of the model comparison made to evaluate whether our remodeled data fitted the PCM or the RSM better. In both cases, the standardized residuals were again close to their expected values. However, Pearson's chi-squared test turned out to be nonsignificant only for the RSM, suggesting an inadequate fit of our data to the PCM. Accordingly, the RSM again was the model of choice.

**Table 3:** Fit statistics of the 3-category PCM and RSM.

**Rasch model**	**Standardized residuals**		**Pearson** χ^**2**^ **test**		**AIC**	**BIC**	**RMSR**	
	***M***	***SD***	χ^2^	***p***			**obs**.	**exp**.
PCM (25 estimated parameters)	0.00	1.03	1,539.21	0.03	2,239.96	2,372.10	0.5543	0.5566
RSM (13 estimated parameters)	0.00	1.01	1,487.17	0.29	2,259.83	2,328.54	0.5568	0.5588

PCM, partial credit model; RSM, rating scale model; AIC, Akaike information criterion; BIC, Bayesian information criterion; RMSR, root mean square residual; obs., observed; exp., expected.

#### 3.2.3 Category effectiveness (RQ 3)

**Figure 4:**
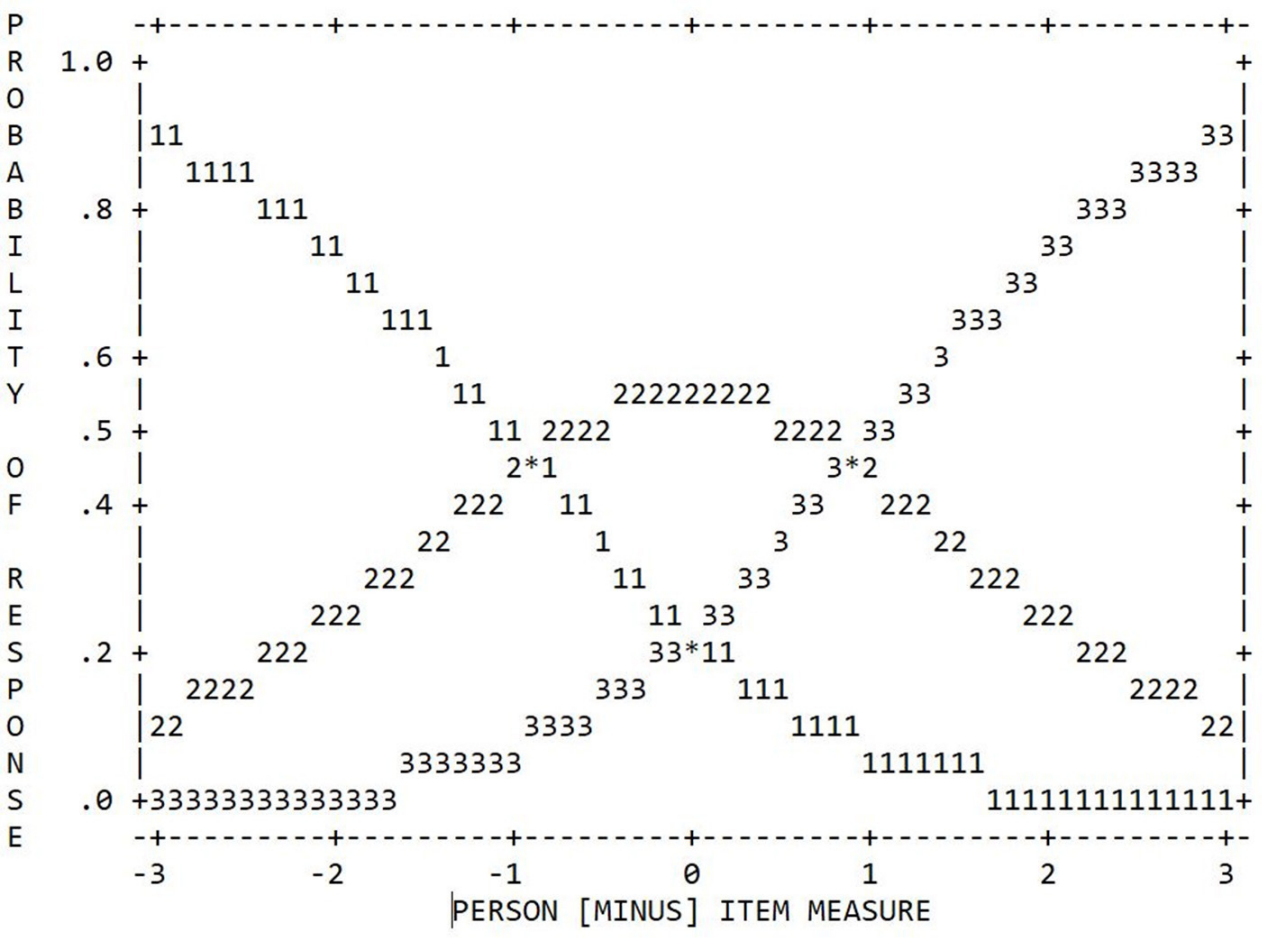
Category probability curves of the 3-category TSR-EBI (RSM); initial sample (*N* = 113).

Our results again showed a sufficient number of 10 observations per category (see Table 4) and a regular distribution pattern (see Figure 4). However, collapsing the categories produced jumps in frequency with increasing category scores. With respect to the coherence, the C→M index of Category 1 was still below the level of 40.0%, but, this time, all other indices were above this cut-off value. Regarding the average measurements and step difficulties, we again found a monotonic increase with increasing category score. However, collapsing the categories caused the point-biserial correlations to be regularly ordered and to have distinguishable levels this time. In addition, the advance in step difficulty for the 3-category model was 1.80 logits and thus within the intended range. Finally, regarding the INFIT and OUTFIT MNSQ values associated with the responses in each category, values close to the expected values of 1.00 were obtained for all categories this time.

**Table 4:** Category statistics of the 3-category TSR-EBI.

**Rating scale category**	**Observed count**	**Average measure**	**MNSQ**		**Step difficulty**	**Coherence**		***r*_pbis_**
			**INFIT**	**OUTFIT**		**M**→**C**	**C**→**M**	
1	215	−0.62	0.99	1.10	n.a.	75.0%	26.0%	−0.37
2	541	0.58	0.99	0.97	−0.90	50.0%	71.0%	−0.15
3	716	1.79	1.00	1.00	0.90	75.0%	66.0%	0.40

MNSQ, mean square; M→C, measure implies category; C→M, category implies measure; r_pbis_, point-biserial correlation. The underlying model is the rating scale model (RSM). All model parameters were estimated using conditional maximum-likelihood estimation (CMLE).

#### 3.2.4 Item fit (RQ 4)

Table 5 provides an overview of the relevant item parameters. All item-related INFIT MNSQ values showed acceptable values (ranging from 0.72 to 1.40). The same was true for the OUTFIT MNSQ values, with a range of 0.77–1.46. The point-biserial correlations were positive for all items, showing that the item scores and the latent variable were oriented in the same direction. Regarding the discrimination, four items showed lower values than expected by the model, with by far the smallest values resulting for Items 12 and 10.

**Table 5:** Item statistics of the 3-category TSR-EBI.

**Item no**.	**Score**	**Measure**		**MNSQ**		***r*_pbis_**	**Discrimination**
		**WLE**	**SE**	**INFIT**	**OUTFIT**		
1	259	0.23	0.16	1.05	1.13	0.38	0.85
2	228	0.89	0.16	1.03	0.99	0.48	1.00
3	215	1.18	0.16	0.92	0.94	0.47	1.07
4	262	0.10	0.16	1.04	1.33	0.41	0.83
5	254	0.19	0.16	0.72	0.77	0.53	1.27
6	288	−0.52	0.18	0.91	0.77	0.46	1.16
7	268	−0.04	0.17	0.98	1.07	0.55	1.05
8	290	−0.66	0.18	0.91	0.81	0.46	1.09
9	236	0.75	0.16	1.03	0.95	0.52	1.13
10	285	−0.43	0.17	1.35	1.46	0.40	0.76
11	300	−1.00	0.20	0.86	0.94	0.45	1.06
12	291	−0.69	0.18	1.40	1.33	0.24	0.63
13	269	−0.01	0.16	0.80	0.82	0.53	1.22

WLE, weighted likelihood estimation; SE, standard error of estimate; MNSQ, mean square; r_pbis_, point-biserial correlation. The underlying model is the rating scale model. All model parameters were estimated using conditional maximum-likelihood estimation (CMLE).

Overall, the Wright map (see Supplementary Figure S1) showed that the items were located only at the low and medium but not at the higher trait levels. In addition, there were four overlaps involving Items 1 and 5, 7 and 13, 6 and 10, and 8 and 12. Finally, we found two larger item gaps, between Items 9 and 1/5, and between Items 7/13 and 6/10.

#### 3.2.5 Reliability (RQ 5)

The person reliability was 0.77 and was thus slightly below the intended value of ≥0.80. However, this finding corresponded with the Wright map in that both indicated that the number of items may have been too small to capture all of the trait variability. The item reliability, on the other hand, was satisfactory with a value of 0.93.

### 3.3 Cross-validation (RQ 6)

After having checked all these quality criteria, we evaluated the empirical usefulness of the 3-category TSR-EBI by administering it to our cross-validation sample. For this data, we performed the same statistical analyses as in the first round of validation (RQ 1–RQ 5).

#### 3.3.1 Assumptions of Rasch measurement (RQ 1)

For both the RSM and the PCM, the total variance explained by the model was somewhat below the values found for the 3-category structure in the first round of validation. For the RSM, it was 2.7% less, resulting in 36.8% (19.1% attributable to persons; 17.7% attributable to item difficulties); for the PCM it was 3.7% less, resulting in 36.4% (19.7% attributable to persons; 16.7% attributable to item difficulties). However, both values were again close to the Rasch model predictions of 36.9% (RSM) and 36.3% (PCM), respectively. Regarding the eigenvalue of the first residual variance contrast, both models were again below the threshold of 2.00 with values of 1.85 (RSM) and 1.77 (PCM). In terms of the total variance, 9.0% (RSM) and 8.6% (PCM) were explained by this first residual variance contrast, which is comparable to the finding for the 3-category structure in the first round of validation. Accordingly, the assumption of unidimensionality was again met for both models. With regard to the correlations between the standardized residuals, for both models, the mean was *r* = −0.08 for the third time. The highest positive correlations were considerably lower than those found in the first round of validation, reaching values of *r* = 0.18 in the case of both the RSM (between Items 7 and 10) and the PCM (between Items 2 and 5). Thus, we were also able to assume local independence for both models. Additionally, van-den-Wollenberg's *Q*_1_ statistic again turned out to be nonsignificant for both the RSM (*Q*_1_ = 159.89, *p* = 0.66) and the PCM (*Q*_1_ = 150.08, *p* = 0.84), suggesting that specific objectivity could also be assumed. Consequently, again, both the RSM and the PCM met all three basic assumptions of Rasch measurement.

#### 3.3.2 Model fit (RQ 2)

Table 6 shows the results of the model comparison made to evaluate whether our data fitted the PCM or the RSM better. In both cases, the standardized residuals were close to their expected values and Pearson's chi-squared test turned out to be nonsignificant, suggesting an adequate fit of the data to each of the two models. In terms of information criteria, the AIC favored the PCM, whereas the BIC favored the RSM. The RMSRs in both cases were very similar and close to those predicted by each model. Therefore, similar to the 5-category scale, we again had two equally fitting models, which is why we gave preference to the RSM, in line with the criterion of parsimony.

**Table 6:** Fit statistics of the 3-category PCM and RSM.

**Rasch model**	**Standardized residuals**		**Pearson** χ^**2**^ **test**		**AIC**	**BIC**	**RMSR**	
	***M***	***SD***	χ^2^	***p***			**obs**.	**exp**.
PCM (24 estimated parameters)	0.00	1.00	951.08	0.37	1,427.11	1,543.94	0.5114	0.5121
RSM (13 estimated parameters)	0.00	0.99	948.00	0.49	1,436.05	1,499.33	0.5156	0.5157

PCM, partial credit model; RSM, rating scale model; AIC, Akaike information criterion; BIC, Bayesian information criterion; RMSR, root mean square residual; obs., observed; exp., expected. For the PCM, the number of estimated parameters is different to that of the first round of validation because there were no missing data in the cross-validation.

#### 3.3.3 Category effectiveness (RQ 3)

**Figure 5:**
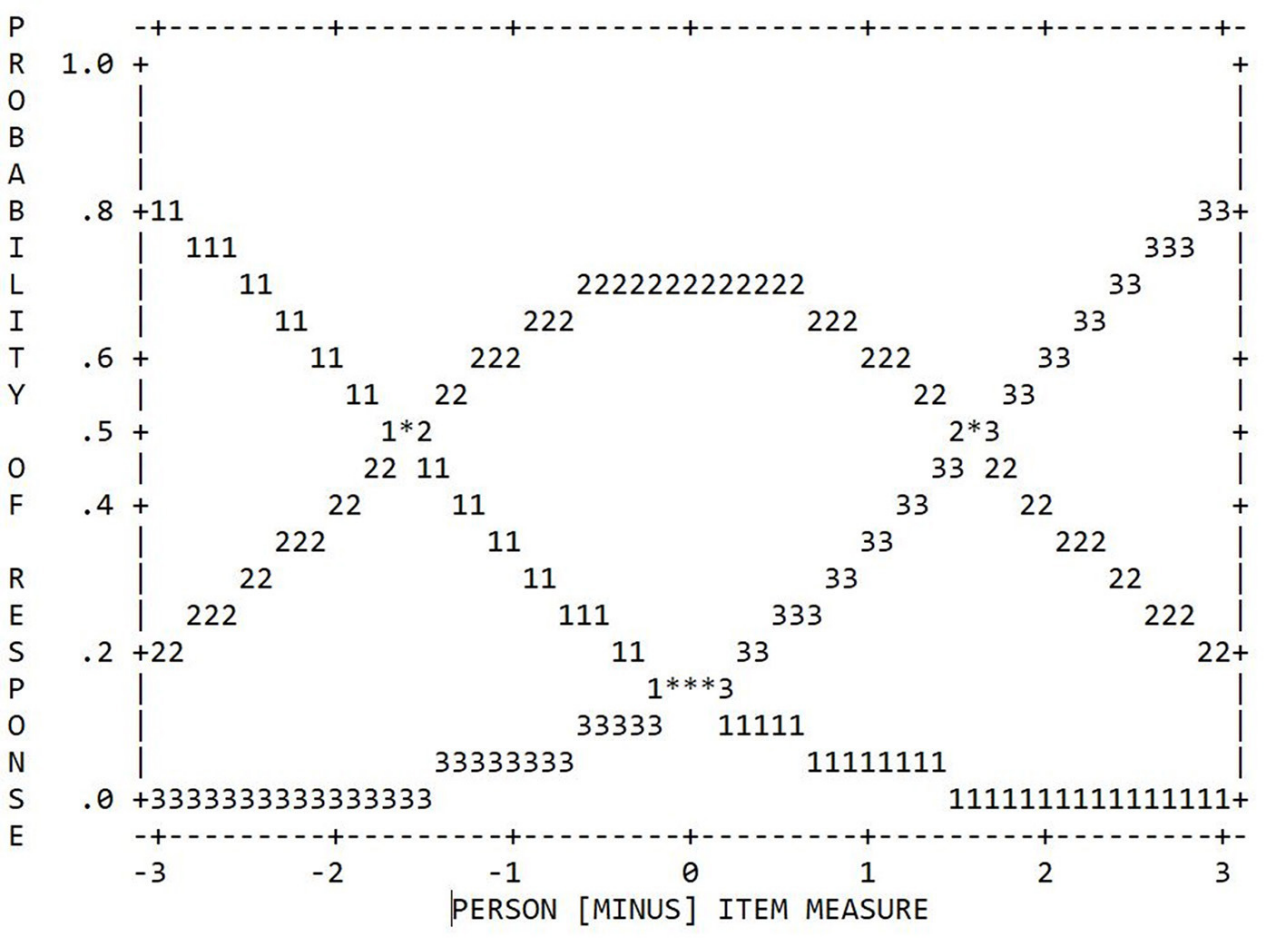
Category probability curves of the 3-category TSR-EBI (RSM); cross-validation sample (*N* = 74).

Our results again showed a sufficient number of 10 observations per category (see Table 7) and a regular distribution pattern (see Figure 5). In the cross-validation, there was no longer a jump in frequency with increasing category scores. Instead, the highest response frequency was found in Category 2, closely followed by Category 3, while Category 1 continued to have the lowest response frequency. Accordingly, the C→M index of Category 1 was still below the level of 40.0%, whereas all other indices were above this cut-off value. Regarding the average measurements, the step difficulties, and the point-biserial correlations, we again found a monotonic increase with increasing category score. The increase in step difficulty was 3.20 logits, which was a significant increase compared to the first round of validation. However, it was still within the intended range. Finally, regarding the INFIT and OUTFIT MNSQ values associated with the responses in each category, again, values close to the expected values of 1.00 were obtained for all categories.

**Table 7:** Category statistics of the 3-category TSR-EBI.

**Rating scale category**	**Observed count**	**Average measure**	**MNSQ**		**Step difficulty**	**Coherence**		***r*_pbis_**
			**INFIT**	**OUTFIT**		**M**→**C**	**C**→**M**	
1	111	−0.81	1.01	1.01	n.a.	64%	26.0%	−0.31
2	494	0.60	0.96	0.93	−1.60	64%	80.0%	−0.17
3	356	1.92	1.02	1.02	1.60	70%	59.0%	0.39

MNSQ, mean square; M→C, measure implies category; C→M, category implies measure; r_pbis_, point-biserial correlation. The underlying model is the rating scale model (RSM). Model parameters were estimated using conditional maximum-likelihood estimation (CMLE).

#### 3.3.4 Item fit (RQ 4)

Table 8 provides an overview of the relevant item parameters. All item-related INFIT MNSQ values again showed acceptable values (ranging from 0.70 to 1.37). The same was true for the OUTFIT MNSQ values, with a range of 0.64–1.35. The point-biserial correlations were positive for all items, showing that the item scores and the latent variable were oriented in the same direction. Regarding the discrimination, six items showed lower values than expected by the model, with by far the smallest values resulting for Items 4 and 9 this time.

**Table 8:** Item statistics of the 3-category TSR-EBI.

**Item no**.	**Score**	**Measure**		**MNSQ**		***r*_pbis_**	**Discrimination**
		**WLE**	**SE**	**INFIT**	**OUTFIT**		
1	159	0.37	0.22	0.89	0.89	0.42	1.13
2	156	0.51	0.22	0.83	0.85	0.31	1.17
3	153	0.64	0.22	1.16	1.16	0.31	0.77
4	164	0.15	0.22	1.37	1.35	0.35	0.53
5	170	−0.12	0.22	0.76	0.72	0.59	1.35
6	178	−0.50	0.23	0.94	0.88	0.60	1.13
7	171	−0.17	0.22	0.90	0.91	0.59	1.14
8	173	−0.35	0.22	1.06	1.06	0.34	0.90
9	137	1.34	0.22	1.32	1.28	0.42	0.63
10	160	0.33	0.22	0.88	0.86	0.66	1.20
11	192	−1.25	0.25	0.70	0.64	0.55	1.34
12	184	−0.81	0.24	1.15	1.18	0.36	0.79
13	170	−0.12	0.22	0.97	1.02	0.39	0.99

WLE, weighted likelihood estimation; SE, standard error of estimate; MNSQ, mean square; r_pbis_, point-biserial correlation. The underlying model is the rating scale model. All model parameters were estimated using conditional maximum-likelihood estimation (CMLE).

The Wright map (see Supplementary Figure S2) shows that most of the item difficulty hierarchy could be replicated, despite minor differences in the positions of individual items. However, there were still no items located on the higher trait levels, although the map showed an overall increased item variability compared to the first round of validation. The number of overlaps decreased from four to only two, but this time there was a cluster of three (instead of only two) items. The overlaps involved Items 1 and 10 as well as 13, 5, and 7. Finally, we again found two larger item gaps, between Item 9 and Item 3 and between Item 12 and Item 11.

#### 3.3.5 Reliability (RQ 5)

The person reliability was 0.79 and was thus somewhat higher than in the first round of validation but was still slightly below the intended value of ≥0.80. The item reliability, on the other hand, was 0.89 this time and, thus, had decreased slightly. Nevertheless, this value was still acceptable.

### 3.4 Combined sample from the first round of validation and the cross-validation (RQ 7)

To collect validity evidence from the relations to other variables, we performed a known-groups comparison among pre-service teachers studying different combinations of teaching subjects.

**Figure 6:**
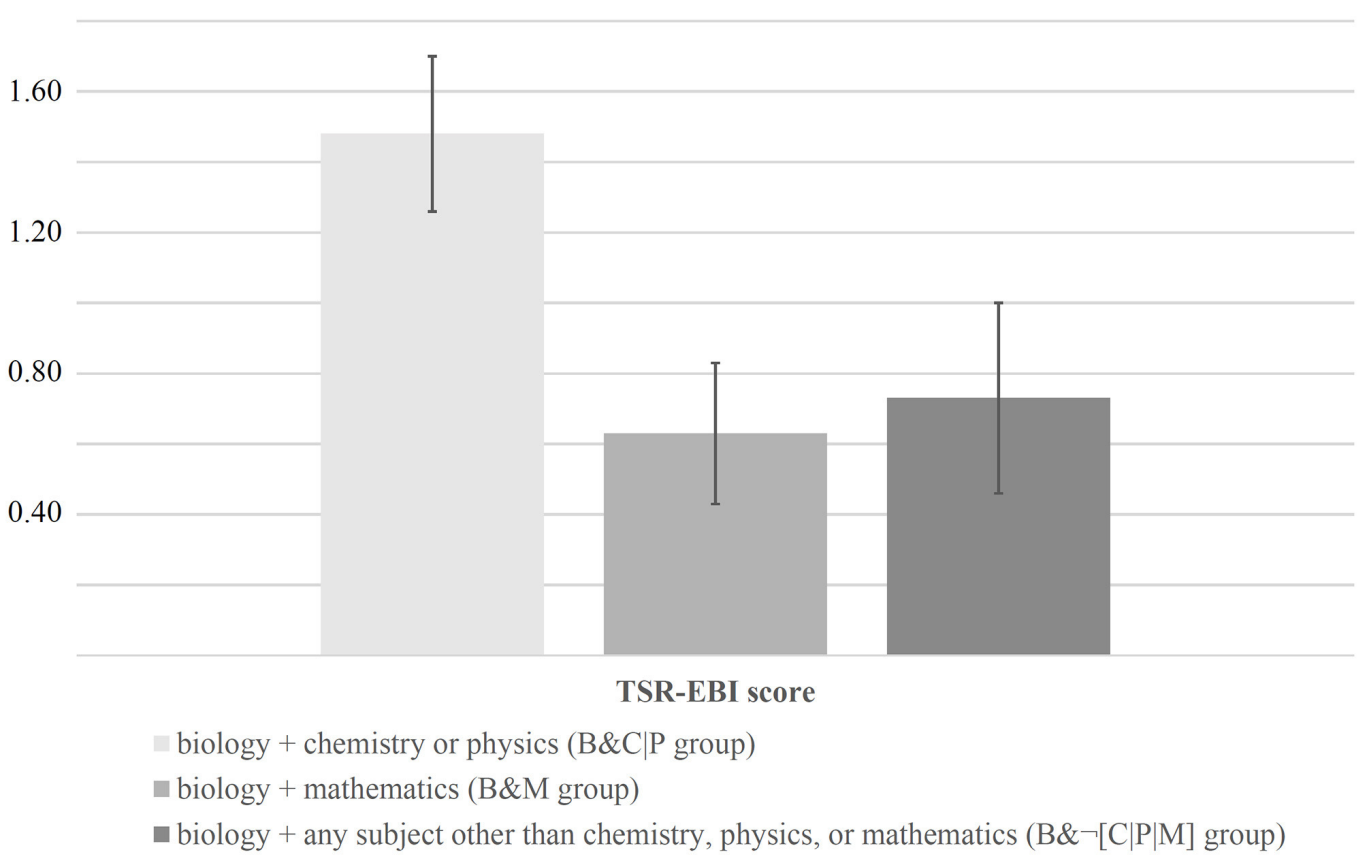
TSR-EBI scores of pre-service teachers studying different combinations of teaching subjects.

Figure 6 shows that the B&C|P group achieved the highest TSR-EBI scores (*M* = 1.48, *SD* = 1.07). In contrast, the B&¬[C|P|M] group scored, on average, only half as high (*M* = 0.73, *SD* = 1.29). The lowest scores (*M* = 0.63, *SD* = 0.95) were achieved by the B&M group.

The univariate ANOVA results (one ANOVA for each random sample, making a total of 10 analyses) consistently showed the overall group differences to be statistically significant: $\bar{F}$(2, 66) = 4.09 (*F*_min_ = 3.36, *F*_max_ = 4.67), $\bar{p}$ = 0.02 (*p*_min_ = 0.01, *p*_max_ = 0.04), $\bar{d}$ = 0.70. Subsequent Tukey *post-hoc* tests that we conducted to localize the between-subjects effects identified the difference between the B&C|P and the B&M groups as statistically significant (*p* < 0.05) in all cases (10 out of 10): $\bar{p}$ = 0.03. In contrast, between the B&C|P and the B&¬[C|P|M] groups, statistically significant differences existed only in five out of 10 cases: $\bar{p}$ = 0.09. Finally, no statistically significant differences (0 out of 10 cases) existed between the B&M and the B&¬[C|P|M] groups: $\bar{p}$ = 0.88.

## 4 Discussion

In our study, we adapted STEBI items (Riggs and Enochs, 1990) to the task of teaching SR and we collected evidence for the psychometric quality of the newly developed TSR-EBI using Rasch measurement. Already during the test development, we addressed important aspects concerning validity of content (American Educational Research Association et al., 2014). In the course of an analysis of the TSR-EBI's internal structure and its relations to other variables (see Section 3), it was possible to collect evidence of its construct and criterion validity, its reliability, and the replicability of its results. Nevertheless, some findings also suggest that the scale could possibly be further optimized, especially with regard to its item selection. Against this background, our research questions can be answered as follows.

The empirical TSR-EBI data matrix met the general assumptions of Rasch measurement, both in the case of each of the two Rasch models considered and with respect to both rating scale category structures (RQ 1). Therefore, the chosen analyses of the TSR-EBI's internal structure were applicable and interpretable. When comparing the two Rasch models, the RSM was always shown to be superior to the PCM, either because of its better fit to the data or, given the identical goodness of fit, because of its parsimony (RQ 2). However, this result was also desirable, as the TSR-EBI's items are obviously intended to share the same Likert agreement. Regarding the number of rating scale categories, a 3-category structure was superior to a 5-category structure in our analyses (RQ 3). By collapsing the exterior categories (1/2 and 4/5), it was possible to improve some indicators of measurement quality that had not proved satisfactory when a 5-category structure was assumed. Given the almost constant variance explanation (when considering the RSM), both the eigenvalue of the first residual contrast and the level of correlations among the standardized residuals decreased, which means that the fit to the basic assumptions of Rasch measurement improved considerably. Regarding the category effectiveness, collapsing the exterior categories caused an improvement of the coherence indices, a regular ordering of point-biserial correlations, an increase in the advance in step difficulty, and an improvement of the INFIT and OUTFIT MNSQ values associated with the responses in each category. Only the C→M index of Category 1 was still below the cut-off value of 40.0%, even with the 3-category structure. However, this could also be a result of the item selection, for which the Wright map (see Supplementary Figure S1) offered potential for improvement despite acceptable INFIT and OUTFIT MNSQ values, point-biserial correlations, and discrimination values (RQ 4). Apart from that, however, the person and item reliability of the scale proved to be acceptable (RQ 5).

More concrete suggestions for the further improvement of the scale were then found during the cross-validation. Here, first of all, it became clear that the results previously found for the 3-category scale were generally replicable across a new (but comparable) sample (RQ 6), which clearly supports the TSR-EBI's psychometric quality. The slightly reduced variance explanation (when considering the RSM) can plausibly be explained by sample-dependent variations (Cohen et al., 2021). While the category effectiveness and item statistics again turned out to be acceptable, the Wright map (see Supplementary Figure S2) once again showed possible starting points for optimization. Although the item difficulty hierarchy was replicated to a substantial extent, which again indicates the good item reliability of the scale, the TSR-EBI could possibly benefit from having redundant items removed, item gaps filled, and more difficult items added. However, as only Items 7 and 13 were found to be identically redundant in both data collections, it would probably be reasonable to start any further revision with the removal or reformulation of one of these two items. Item 7 refers to difficulties in explaining SR to students; Item 13 relates to difficulties in making students more enthusiastic about SR (see section Supplementary Information on the Wording of the TSR-EBI Items in Supplementary material). Obviously, the use of both items does not offer any additional predictive value in the assessment of pre-service teachers' self-efficacy. Therefore, it might be appropriate to revise one of them. In addition, further items of higher difficulty could already be added, as this revision step was also suggested by the findings of both data collections. It is highly probable that the TSR-EBI's precision could be improved by such an addition. Likewise, such a revision step may result in an additional improvement of the person reliability. However, further revision steps would not be worth considering before one or more additional rounds of data collections are completed, as the remaining results (e.g., on item gaps) were found to be variable between our samples.

In terms of test-criterion relationships, the scale was clearly shown to be able to discriminate between groups that are assumed to have different levels of self-efficacy regarding teaching SR (RQ 7). As expected, the group of pre-service teachers who combined two science subjects achieved significantly higher TSR-EBI scores than the groups with other subject combinations. Hence, our finding is in line with, for example, the study by Welter et al. (2022), which also suggested synergistic effects of specific combinations of pre-service teachers' majors on the development of their professional competence. However, whether these higher TSR-EBI scores are actually reflected in improved long-term teaching success must first be investigated by means of appropriate longitudinal assessments before any recommendations for action can be derived.

The fact that the B&M group nominally performed the worst may seem somewhat counterintuitive at first glance, but it can be plausibly explained in two ways: first, teaching SR is not an explicit goal of mathematics education in Germany (Kultusministerkonferenz, 2004a,b,c), which means that it cannot be expected that pre-service mathematics teachers are prepared for this task during their university studies. Second, in addition to the lack of learning opportunities, rather person-related factors could also be responsible for the finding that the B&M group achieved almost identical TSR-EBI scores as the B&¬[C|P|M] group. Indeed, the well-known general negative effect of mathematics as a second subject might have become visible here. The high complexity associated with a university degree in mathematics regularly leads to students being overwhelmed and sometimes even dropping out of their studies (Hoyles et al., 2001; Neumann et al., 2021a). For teacher education in particular, studies have reported negative effects of mathematics as a second subject on the development of pre-service teachers' professional competence (even for competence aspects related to the subject studied besides mathematics; e.g., Neumann et al., 2021b).

### 4.1 Study limitations and prospects for future research

Although we were able to collect some evidence for the TSR-EBI's psychometric quality, our results must also be evaluated in terms of our study's limitations.

Our sample sizes of *N* = 114 (first round of validation) and *N* = 74 (cross-validation) were comparatively small. Although both samples were larger than the minimum sample size of 50 suggested by Linacre (1994) and the high item reliability indicated a sufficiently large sample (Boone et al., 2014), it might be possible to obtain more precise IRT-based parameter estimates with a larger number of participants (Hambleton, 1989). Larger samples could also allow for subgroup stratification and, thus, the identification of potential differential item functioning (DIF). For example, an interesting question would be whether systematically different TSR-EBI scores can be expected for pre-service teachers of different genders. In the context of explanations for gender gaps in STEM fields, it has been shown many times that female participants score lower on self-efficacy variables, despite having similar levels of prior achievement (e.g., Huang, 2013; Zander et al., 2020; Robinson et al., 2022). The exclusion of a relevant DIF with respect to the respondents' gender would therefore be another important indicator of validity (American Educational Research Association et al., 2014; Gómez-Benito et al., 2018).

In our study, all participants studied the subject of biology and were recruited from just two German universities, which certainly limits the results' external validity (Cohen et al., 2021). The decision to include only pre-service biology teachers had pragmatic reasons related to the fact that the colleagues who recruited the samples in their university lectures belong to the field of biology education. In our opinion, however, the TSR-EBI could probably be easily adapted for use with, for example, chemistry or physics pre-service teachers. For this purpose, the term “biology class” (see section Supplementary Information on the Wording of the TSR-EBI Items in Supplementary material) in Items 1, 2, 3, and 6 would just have to be specified for other scientific disciplines. We welcome other researchers to take up this suggestion. Only with the help of follow-up studies with more diverse samples and conducted at more locations can a reliable statement about the scope of the test score interpretation be made (American Educational Research Association et al., 2014).

Another limitation concerns our approach of collapsing categories. Although our results suggested that it was reasonable to collapse Categories 1 and 2 as well as Categories 4 and 5 into one category each, one might well ask what an alternative modeling of the category structure would have resulted in. For example, we could have combined only Categories 1 and 2 at first, because Category 5 was found to perform considerably better in comparison. However, such a revision, which would have implied a change from an odd to an even number of categories, would necessarily have resulted in the elimination of the neutral category. This would have changed the meaning of all the categories, making it impossible to remodel the category structure with existing data. Instead, it would have been necessary to present the new scale to a new sample (Linacre, 2022). If such a further round of data collection had revealed a need for further optimization (e.g., regarding Category 5), a third round of data collection would have been necessary, and so on. For efficiency reasons, this alternative therefore seemed less favorable than the one we chose, all the more so as the results for Category 5 turned out to be comparatively unsatisfactory. Nevertheless, it cannot be ruled out that data obtained from follow-up studies will suggest a different approach, which could then also lead to different overall conclusions about the psychometric quality of the TSR-EBI.

Another important limitation relates to the limited practical teaching experience of pre-service teachers. They were still completing the academic part of their teacher training, that is, they had only been able to gain teaching experience in the context of short-term internships in schools so far. The extent to which their self-efficacy expectations will prove stable after the transition to practical professional life can therefore only be answered by future longitudinal assessments.

However, apart from this, it would also be interesting to collect TSR-EBI data in a sample of in-service teachers to compare their person parameters with those of the pre-service teachers in our study. Looking at the Wright maps (see Supplementary Figures S1, S2), it is noticeable, especially for the participants in the first round of validation, that there were significantly more positive than negative self-efficacy ratings in relation to the mean value. This might be due to an unrealistic overestimation of the participants' own abilities due to their lack of practical teaching experience. A study by Settlage et al. (2009) also found that pre-service teachers were highly confident in their ability to effectively teach science despite being inexperienced and having limited formal preparation. However, in our study, this finding could possibly also be confounded either with habitual self-efficacy or with overall superior cognitive abilities (or both). Based on our analyses of RQ 7, it can be assumed that high TSR-EBI scores are found more often among those pre-service teachers who study two science subjects. This combination of subjects is chosen quite rarely, not least because many students find the natural sciences comparatively complicated and abstract (e.g., Cuff, 2017; Shirazi, 2017). Accordingly, students with this subject combination may also generally be more confident in their own abilities, possibly because they generally have high cognitive abilities and could trust in them in previous performance situations. The TSR-EBI's discriminant validity regarding such constructs may therefore need to be tested in follow-up studies, for example, by correlating TSR-EBI scores with those of the Core Self-Evaluations Scale (Judge et al., 2006) and with a cognitive abilities test (e.g., the Wechsler Adult Intelligence Scale; Wechsler, 2008).

Finally, the comparatively high self-efficacy scores could also (at least in part) be the result of socially desirable response behavior. Despite assuring maximum anonymity and waiving a collection of personal data to a large extent, the recruitment of participants by lecturers in university courses could have induced socially desirable response behavior. Unfortunately, due to limited time and personnel resources, we were unable to acquire the samples in any other way. Therefore, to make a valid estimate of potential construct-irrelevant variance due to socially desirable response behavior, two approaches would be conceivable in follow-up studies. Either, if participants continue to be recruited by university lecturers, an additional scale could be used to assess socially desirable response behavior (e.g., the Social Desirability Scale-17; Stöber, 2001), or an attempt could be made to obtain samples outside of university courses, for example, by asking student councils or other student representatives to share a link to an online survey with their members. However, this inclusion of third parties would on the other hand be associated with factors that are beyond the researcher's control, possibly resulting in a significantly prolonged recruitment period and a higher level of effort to achieve an adequate sample size.

## 5 Conclusion

The first aim of our study was to provide initial evidence for the TSR-EBI's psychometric quality. We believe that we have successfully achieved this aim, as most of our results support the psychometric quality of the TSR-EBI. The remaining results may be useful for its improvement, which, in our opinion, should focus on removing redundant items and developing more difficult ones (see first part of Section 4). Furthermore, our results make it possible to gain perspectives on the design of future studies that could go beyond simply removing our study's limitations. These might address, for example, an examination of the TSR-EBI's retest reliability or aspects of its predictive and discriminant validity (see Section 4.1). However, in addition to these psychometric issues, we also hope that the TSR-EBI can be useful for practical purposes in the future. The instrument has the potential to contribute to a better understanding of (pre-service) teachers' self-efficacy regarding teaching SR. Such understanding, in turn, is important for the implementation of effective training in university teacher education and for the development of professional development programs. For example, the TSR-EBI is currently used to evaluate the effect of a professional development program to foster science teachers' professional competence related to scientific reasoning (Sannert and Krell, 2023).

Finally, we hope that we have also succeeded in fulfilling the second aim of our study, which was to provide comprehensible insights to those readers who are not yet familiar with the use of Rasch measurement in validating rating scales. Hence, researchers that are aiming to collect validity evidence for other rating scale instruments might use our study as an illustrative example how to realize guidelines from the methodological literature, such as Linacre (2002). In any case, however, we believe that we have been able to show that validation is not a routine procedure and, hence, is not something that can be done casually. In fact, as Kane (1992) and Messick (1995) pointed out, it is an ongoing process that is based on numerous considerations and likely involves multiple rounds of revisions and data collections.

## Data availability statement

The raw data supporting the conclusions of this article will be made available by the authors, without undue reservation.

## Ethics statement

The studies involving humans were approved by the Local Ethics Committee of the Leibniz Institute for Science and Mathematics Education, Kiel, Germany (ID: 2021_KR43). The studies were conducted in accordance with the local legislation and institutional requirements. The participants provided their written informed consent to participate in this study.

## Author contributions

VW: Conceptualization, Data curation, Formal analysis, Methodology, Software, Validation, Visualization, Writing—original draft, Writing—review & editing. MD-G: Funding acquisition, Writing—review & editing. LG: Funding acquisition, Investigation, Writing—review & editing. MK: Conceptualization, Funding acquisition, Investigation, Project administration, Resources, Supervision, Writing—original draft, Writing—review & editing.

## Footnotes

^1^Basically, it would additionally be possible to convert the INFIT and OUTFIT MNSQ values to an approximately normalized *t*-statistic using a Wilson-Hilferty transformation (Wilson and Hilferty, 1931). However, we do not report such *t*-values because they can be ignored if the MNSQ values are between 0.50 and 1.50 (Linacre, 2022), which was the case in this study (see Section 3).

## References

[B1] Akaike H. (1974). “A new look at the statistical model identification,” in Selected Papers of Hirotugu Akaike, eds Parzen E. Tanabe K. Kitagawa G. (New York, NY: Springer New York), 215–222. 10.1007/978-1-4612-1694-0_16

[B2] Al Sultan A. A. (2020). Investigating preservice elementary teachers' subject-specific self-efficacy in teaching science. EURASIA J. Math. Sci Tech. Ed. 16:em1843. 10.29333/ejmste/7801

[B3] American Educational Research Association American Psychological Association, and National Council on Measurement in Education. (2014). Standards for Educational and Psychological Testing. Washington, DC: American Educational Research Association.

[B4] Andersen A. M. Dragsted S. Evans R. H. Sørensen H. (2004). The relationship between changes in teachers' self-efficacy beliefs and the science teaching environment of Danish first-year elementary teachers. J. Sci. Teacher Educ. 15, 25–38. 10.1023/B:JSTE.0000031461.68912.3d

[B5] Andrich D. (1978). A rating formulation for ordered response categories. Psychometrika 43, 561–573. 10.1007/BF02293814

[B6] Andrich D. (2004). Controversy and the Rasch model: a characteristic of incompatible paradigms? Med. Care 42, 7–16. 10.1097/01.mlr.0000103528.48582.7c 14707751

[B7] Andrich D. Marais I. (2019). A Course in Rasch Measurement Theory: Measuring in the Educational, Social and Health Sciences. Singapore: Springer Nature Singapore. 10.1007/978-981-13-7496-8

[B8] Ballantyne J. Retell J. (2020). Teaching careers: exploring links between well-being, burnout, self-efficacy and praxis shock. Front. Psychol. 10:2255. 10.3389/fpsyg.2019.02255 32132940 PMC7040245

[B9] Bandura A. (1977). Self-efficacy: toward a unifying theory of behavioral change. Psychol. Rev. 84, 191–215. 10.1037/0033-295X.84.2.191 847061

[B10] Bandura A. (1986). Social Foundations of Thought and Action: A Social Cognitive Theory. Englewood Cliffs, NJ: Prentice-Hall.

[B11] Bandura A. (1994). “Self-efficacy” in Encyclopedia of Human Behavior, ed. V.S. Ramachandran (New York, NY: Academic Press), 71–81.

[B12] Bandura A. (1997). Self-Efficacy: The Exercise of Control. New York, NY: W.H. Freeman/Times Books/Henry Holt and Co.

[B13] Bandura A. (2001). Social cognitive theory: an agentic perspective. Annu. Rev. Psychol. 52, 1–26. 10.1146/annurev.psych.52.1.1 11148297

[B14] Bandura A. (2006). “Guide for constructing self-efficacy scales” in Self-Efficacy Beliefs of Adolescents, eds Pajares F. Urdan T. (Greenwich, CT: Information Age Publishing), 307–337.

[B15] Barton M. A. Lord F. M. (1981). An upper asymptote for the three-parameter logistic item-response model. ETS Res. Rep. Ser. 1981, i−8. 10.1002/j.2333-8504.1981.tb01255.x

[B16] Birnbaum A. (1968). “Some latent trait models and their use in inferring an examinee's ability” in Statistical Theories of Mental Test Scores, eds Lord F.M. Novick M.R. (Reading, MA: Addison-Wesley), 19–20.

[B17] Blömeke S. Jentsch A. Ross N. Kaiser G. König J. (2022). Opening up the black box: teacher competence, instructional quality, and students' learning progress. Learn. Instr. 79:101600. 10.1016/j.learninstruc.2022.101600

[B18] Bond T. G. Yan Z. Heene M. (2020). Applying the Rasch Model: Fundamental Measurement in the Human Sciences. New York, NY: Routledge. 10.4324/9780429030499

[B19] Bong M. (1997). Generality of academic self-efficacy judgments: evidence of hierarchical relations. J. Educ. Psychol. 89, 696–709. 10.1037/0022-0663.89.4.696

[B20] Bong M. Skaalvik E. M. (2003). Academic self-concept and self-efficacy: How different are they really? Educ. Psychol. Rev. 15, 1–40. 10.1023/A:1021302408382

[B21] Boone W. J. Staver J. R. Yale M. S. (2014). “Person reliability, item reliability, and more” in Rasch Analysis in the Human Sciences, eds Boone W. J. Staver J. R. Yale M. S. (Dordrecht: Springer Netherlands), 217–234. 10.1007/978-94-007-6857-4_10

[B22] Cannon J. R. Scharmann L. C. (1996). Influence of a cooperative early field experience on preservice elementary teachers' science self-efficacy. Sci. Educ. 80, 419–436. 10.1002/(SICI)1098-237X(199607)80:4&lt;419::AID-SCE3&gt;3.0.CO;2-G

[B23] Chambers C. R. Walpole M. Outlaw N. (2016). The influence of math self-efficacy on the college enrollments of young black women. *J*. Negro Educ. 85, 302–315. 10.7709/jnegroeducation.85.3.0302

[B24] Chowdhury T. B. M. Holbrook J. Rannikmäe M. (2020). Socioscientific issues within science education and their role in promoting the desired citizenry. Sci. Educ. Int. 31, 203–208. 10.33828/sei.v31.i2.10

[B25] Clinton J. Koelle M. Aston R. (2018). Investigating the Key Characteristics of Effective Teachers: A Systematic Review. Available online at: https://melbourne.figshare.com/articles/online_resource/Establishing_the_key_determinants_of_effective_teaching_a_systematic_review/6682484/1 (accessed April 10, 2023).

[B26] Cohen R. J. Schneider W. J. Tobin R. M. (2021). Psychological Testing and Assessment. New York, NY: McGraw Hill.

[B27] Cuff B. M. (2017). Perceptions of Subject Difficulty and Subject Choices: Are the Two Linked, and if So, How? Ofqual's Strategy, Risk and Research Directorate. Available online at: https://dera.ioe.ac.uk/30159/1/Perceptions_of_subject_difficulty_and_subject_choices.pdf (accessed November 14, 2023).

[B28] Deehan J. (2017). The Science Teaching Efficacy Belief Instruments (STEBI A and B): A Comprehensive Review of Methods and Findings from 25 Years of Science Education Research. Cham: Springer Switzerland. 10.1007/978-3-319-42465-1

[B29] DeMars C. E. (2018). “Classical test theory and item response theory” in The Wiley Handbook of Psychometric Testing, eds Irwing P. Booth T. Hughes D. J. (Hoboken, NJ: John Wiley and Sons), 49–73. 10.1002/9781118489772.ch2

[B30] Enochs L. G. Riggs I. M. (1990). Further development of an elementary science teaching efficacy belief instrument: a preservice elementary scale. Sch. Sci. Math. 90, 694–706. 10.1111/j.1949-8594.1990.tb12048.x

[B31] Enochs L. G. Smith P. L. Huinker D. (2000). Establishing factorial validity of the mathematics teaching efficacy beliefs instrument. Sch. Sci. Math. 100, 194–202. 10.1111/j.1949-8594.2000.tb17256.x

[B32] European Commission (2015). Science Education for Responsible Citizenship. Brussels: EC. https://op.europa.eu/en/publication-detail/-/publication/a1d14fa0-8dbe-11e5-b8b7-01aa75ed71a1 (accessed January 15, 2024).

[B33] Fisher W. P. Jr. (2007). Rating scale instrument quality criteria. Rasch Meas. Trans. 21:1095.

[B34] Gagnier K. M. Holochwost S. J. Fisher K. R. (2021). Spatial thinking in science, technology, engineering, and mathematics: elementary teachers' beliefs, perceptions, and self-efficacy. J. Res. Sci. Teach. 59, 95–126. 10.1002/tea.21722

[B35] Goddard R. D. Hoy W. K. Woolfolk Hoy A. (2000). Collective teacher efficacy: its meaning, measure, and impact on student achievement. Am. Educ. Res. J. 37, 479–507. 10.3102/00028312037002479

[B36] Gómez-Benito J. Sireci S. Padilla J. L. Hidalgo M. D. Benítez I. (2018). Differential item functioning: beyond validity evidence based on internal structure. Psicothema 30, 104–109. 29363478 10.7334/psicothema2017.183

[B37] Haberman S. J. (2004). Joint and conditional maximum likelihood estimation for the Rasch model for binary responses. ETS Res. Rep. Ser. 2004, i−63. 10.1002/j.2333-8504.2004.tb01947.x

[B38] Hambleton R. K. (1989). “Principles and selected applications of item response theory” in Educational Measurement, ed. R. L. Linn (New York, NY: Macmillan Publishing), 147–200.

[B39] Hambleton R. K. Jones R. W. (1993). Comparison of classical test theory and item response theory and their applications to test development. Educ. Meas.: Issues Pract. 12, 38–47. 10.1111/j.1745-3992.1993.tb00543.x

[B40] Heitzmann A. (2002). Fachliche ausbildung durch “disziplinäre vertiefung” [Subject education via “disciplinary deepening”]. Beitr. Lehrerbildung 20, 364–377. 10.36950/bzl.20.3.2002.10241

[B41] Holden M. E. Groulx J. Bloom M. A. Weinburgh M. H. (2011). Assessing teacher self-efficacy through an outdoor professional experience. Elect. J. Sci. Educ. 12, 1–25.

[B42] Honicke T. Broadbent J. (2016). The influence of academic self-efficacy on academic performance: a systematic review. Educ. Res. Rev. 17, 63–84. 10.1016/j.edurev.2015.11.002

[B43] Hoyles C. Newman K. Noss R. (2001). Changing patterns of transition from school to university mathematics. Int. J. Math. Educ. Sci. Technol. 32, 829–845. 10.1080/00207390110067635

[B44] Huang C. (2013). Gender differences in academic self-efficacy: a meta-analysis. Eur. J. Psychol. Educ. 28, 1–35. 10.1007/s10212-011-0097-y

[B45] Judge T. A. Erez A. Bono J. E. Thoresen C. J. (2006). The core self-evaluations scale: development of a measure. Pers. Psychol. 56, 303–331. 10.1111/j.1744-6570.2003.tb00152.x

[B46] Kane M. T. (1992). An argument-based approach to validity. Psychol. Bull. 112, 527–535. 10.1037/0033-2909.112.3.527

[B47] Khan S. Krell M. (2019). Scientific reasoning competencies: a case of preservice teacher education. Can. J. Sci. Math. Technol. Educ. 19, 446–464. 10.1007/s42330-019-00063-9

[B48] Klassen R. M. Usher E. L. (2010). “Self-efficacy in educational settings: recent research and emerging directions” in The Decade Ahead: Theoretical Perspectives on Motivation and Achievement, eds Urdan T.C. Karabenick S.A. (Leeds, England: Emerald Group Publishing Limited), 1–33. 10.1108/S0749-7423(2010)000016A004

[B49] Komboz B. Strobl C. Zeileis A. (2018). Tree-based global model tests for polytomous Rasch models. Educ. Psychol. Meas. 78, 128–166. 10.1177/0013164416664394 29795950 PMC5965621

[B50] Krell M. Khan S. Vergara C. Cofré H. Mathesius S. Krüger D.. (2023). Pre-service science teachers' scientific reasoning competencies: analysing the impact of contributing factors. Res. Sci. Educ. 53, 59–79. 10.1007/s11165-022-10045-x

[B51] Krell M. Mathesius S. van Driel J. Vergara C. Krüger, D. (2020). Assessing scientific reasoning competencies of pre-service science teachers: translating a German multiple-choice instrument into English and Spanish. Int. J. Sci. Ed. 42, 2819–2841. 10.1080/09500693.2020.1837989

[B52] Kultusministerkonferenz (2004a). Bildungsstandards im Fach Biologie für den Mittleren Schulabschluss (Beschluss der Kultusministerkonferenz vom 16.12.2004) [Educational standards in biology for the secondary school diploma (resolution of the Standing Conference of the Ministers of Education and Cultural Affairs of the Länder in the Federal Republic of Germany of 2004 December, 16)]. Available online at: https://www.kmk.org/fileadmin/veroeffentlichungen_beschluesse/2004/2004_12_16-Bildungsstandards-Biologie.pdf (accessed April 10, 2023).

[B53] Kultusministerkonferenz (2004b). Bildungsstandards im Fach Chemie für den Mittleren Schulabschluss (Beschluss der Kultusministerkonferenz vom 16.12.2004) [Educational standards in chemistry for the secondary school diploma (resolution of the Standing Conference of the Ministers of Education and Cultural Affairs of the Länder in the Federal Republic of Germany of 2004 December, 16)]. Available online at: https://www.kmk.org/fileadmin/Dateien/veroeffentlichungen_beschluesse/2004/2004_12_16- Bildungsstandards-Chemie.pdf (accessed April 10, 2023).

[B54] Kultusministerkonferenz (2004c). Bildungsstandards im Fach Physik für den Mittleren Schulabschluss (Beschluss der Kultusministerkonferenz vom 16.12.2004) [Educational standards in physics for the secondary school diploma (resolution of the Standing Conference of the Ministers of Education and Cultural Affairs of the Länder in the Federal Republic of Germany of 2004 December, 16)]. Available online at: https://www.kmk.org/fileadmin/Dateien/veroeffentlichungen_beschluesse/2004/2004_12_16-Bildungsstandards-Physik-Mittleren-SA.pdf (accessed April 10, 2023).

[B55] Lawson A. (2004). The nature and development of scientific reasoning. Int. J. Sci. Math. Educ. 2, 307–338. 10.1007/s10763-004-3224-2

[B56] Lazarides R. Warner L. M. (2020). “Teacher self-efficacy” in Oxford Research Encyclopedia of Education. Available online at: https://oxfordre.com/education/view/10.1093/acrefore/9780190264093.001.0001/acrefore-9780190264093-e-890 (accessed November 14, 2023).

[B57] Lederman J. S. Lederman N. G. Bartels S. Jimenez J. Acosta K. Akubo M.. (2021). International collaborative follow-up investigation of graduating high school students' understandings of the nature of scientific inquiry: is progress Being made? Int. J. Sci. Educ. 43, 991–1016. 10.1080/09500693.2021.1894500

[B58] Lederman J. S. Lederman N. G. Bartels S. Jimenez J. Akubo M. Aly S.. (2019). An international collaborative investigation of beginning seventh grade students' understandings of scientific inquiry: establishing a baseline. J. Res. Sci. Teach. 56, 486–515. 10.1002/tea.21512

[B59] Linacre J. M. (1994). Sample size and item calibration stability. Rasch Meas. Trans. 7:328.

[B60] Linacre J. M. (2002). Optimizing rating scale category effectiveness. J. Appl. Meas. 3, 85–106. 11997586

[B61] Linacre J. M. (2022). Winsteps^^®^^ *Rasch Measurement Computer Program User's Guide (Version 5.2.3)*. Available online at: https://winsteps.com (accessed June 22, 2023).

[B62] Liu X. (2020). Using and Developing Measurement Instruments in Science Education: A Rasch Modeling Approach. Charlotte, NC: Information Age Publishing.

[B63] Lopez A. T. (1998). The item discrimination index: does it work? *Rasch Meas. Trans*. 12:626.

[B64] Lord F. (1952). A Theory of Test Scores. Psychometric Monograph No. 7. Psychometric Corporation. Available online at: https://www.psychometricsociety.org/sites/main/files/file-attachments/mn07.pdf?1576607452 (accessed November 14, 2023).

[B65] MacKinnon N. J. (2015). Self-Esteem and Beyond. London: Palgrave Macmillan. 10.1057/9781137542304

[B66] Mager T. Hein C. (2019). “Mathematics and/as humanities - linking humanistic historical to quantitative approaches” in The Mathematics of Urban Morphology: Modeling and Simulation in Science, Engineering and Technology, ed. L. D'Acci (Cham: Birkhäuser), 523–528. 10.1007/978-3-030-12381-9_27

[B67] Marsh H. W. Pekrun R. Parker P. D. Murayama K. Guo J. Dicke T.. (2019). The murky distinction between self-concept and self-efficacy: beware of lurking jingle-jangle fallacies. J. Educ. Psychol. 111, 331–353. 10.1037/edu0000281

[B68] Masters G. N. (1982). A Rasch model for partial credit scoring. Psychometrika 47, 149–174. 10.1007/BF02296272

[B69] Masters G. N. (1992). Rasch-Andrich thresholds and rasch-thurstone thresholds. Rasch Meas. Trans. 5:191.

[B70] McDonnough J. T. Matkins J. J. (2010). The role of field experience in elementary preservice teachers' self-efficacy and ability to connect research to practice. Sch. Sci. Math. 110, 13–23. 10.1111/j.1949-8594.2009.00003.x

[B71] Menold N. Bogner K. (2016). Design of Rating Scales in Questionnaires. Mannheim: GESIS – Leibniz Institute for the Social Sciences. 10.15465/gesis-sg_en_015

[B72] Menon D. Sadler T. D. (2017). Sources of science teaching self-efficacy for preservice elementary teachers in science content courses. Int. J. Sci. Math. Educ. 16, 835–855. 10.1007/s10763-017-9813-7

[B73] Mesci G. Schwartz R. S. Pleasants B. A.-S. (2020). Enabling factors of preservice science teachers' pedagogical content knowledge for nature of science and nature of scientific inquiry. Sci. Educ. 29, 263–297. 10.1007/s11191-019-00090-w

[B74] Messick S. (1995). Validity of psychological assessment: validation of inferences from persons' responses and performances as scientific inquiry into score meaning. Am. Psychol. 50, 741–749. 10.1037/0003-066X.50.9.741

[B75] Mohamadi F. S. Asadzadeh H. (2012). Testing the mediating role of teachers' self-efficacy beliefs in the relationship between sources of efficacy information and students' achievement. Asia Pac. Educ. Rev. 13, 427–433. 10.1007/s12564-011-9203-8

[B76] Neumann I. Jeschke C. Heinze A. (2021a). First year students' resilience to cope with mathematics exercises in the University Mathematics Studies. J. Math.-Didakt. 42, 307–333. 10.1007/s13138-020-00177-w

[B77] Neumann I. Sorge S. Hoth J. Lindmeier A. Neumann K. Heinze A.. (2021b). Synergy effects in learning? The influence of mathematics as a second subject on teacher students' physics content knowledge. Stud. High. Educ. 46, 2035–2046. 10.1080/03075079.2021.1953335

[B78] Nie Y. Tan G. H. Liau A. K. Lau S. Chua B. L. (2013). The roles of teacher efficacy in instructional innovation: its predictive relations to constructivist and didactic instruction. Educ. Res. Policy Pract. 12, 67–77. 10.1007/s10671-012-9128-y

[B79] OECD (2023). PISA 2025 Science Framework. Available obline at: https://pisa-framework.oecd.org/science-2025/assets/docs/PISA_2025_Science_Framework.pdf (accessed April 10, 2023).

[B80] Oon P.-T. Fan X. (2017). Rasch analysis for psychometric improvement of science attitude rating scales. Int. J. Sci. Educ. 39, 683–700. 10.1080/09500693.2017.1299951

[B81] Osborne J. (2013). The 21st century challenge for science education: assessing scientific reasoning. think. Skills Create. 10, 265–279. 10.1016/j.tsc.2013.07.006

[B82] Pearson K. (1900). On the criterion that a given system of deviations from the probable in the case of a correlated system of variables is such that it can be reasonably supposed to have arisen from random sampling. Philos. Mag. Ser. 5, 157–175. 10.1080/14786440009463897

[B83] Planinic M. Boone W. J. Susac A. Ivanjek L. (2019). Rasch analysis in physics education research: why measurement matters. Phys. Rev. Phys. Educ. Res. 15:020111. 10.1103/PhysRevPhysEducRes.15.020111

[B84] Raîche G. (2005). Critical eigenvalue sizes (variances) in standardized residual principal components analysis. Rasch Meas. Trans. 19:1012.

[B85] Ramey-Gassert L. Shroyer M. G. Staver J. R. (1996). A qualitative study of factors influencing science teaching self-efficacy of elementary level teachers. Sci. Educ. 80, 283–315. 10.1002/(SICI)1098-237X(199606)80:3<283::AID-SCE2>3.0.CO;2-A

[B86] Rasch G. (1960). Probabilistic Model for some Intelligence and Achievement Tests. Copenhagen: Danish Institute for Educational Research.

[B87] Raudenbush S. W. Rowan B. Cheong Y. F. (1992). Contextual effects on the self-perceived efficacy of high school teachers. Sociol. Educ. 65, 150–167. 10.2307/2112680

[B88] Richardson G. M. Liang L. L. (2008). The use of inquiry in the development of preservice teacher efficacy in Mathematics and Science. J. Elementary Sci. Educ. 20, 1–16. 10.1007/BF03174699

[B89] Riggs I. M. Enochs L. G. (1990). Toward the development of an elementary teacher's science teaching efficacy belief instrument. Sci. Educ. 74, 625–637. 10.1002/sce.3730740605

[B90] Robinson K. A. Perez T. White-Levatich A. Linnenbrink-Garcia L. (2022). Gender differences and roles of two science self-efficacy beliefs in predicting post-college outcomes. J. Exp. Educ. 90, 344–363. 10.1080/00220973.2020.1808944 35282472 PMC8916716

[B91] Ross J. A. Bradley Cousins J. Gadalla T. (1996). Within-teacher predictors of teacher efficacy. Teach. Teach. Educ. 12, 385–400. 10.1016/0742-051X(95)00046-M

[B92] Rubeck M. Enochs L. G. (1991). “A path analytic model of variables that influence science and chemistry teaching self-efficacy and outcome expectancy in middle school science teachers,” in Paper Presentation at the 64th Annual Meeting of the National Association for Research in Science Teaching, Lake Geneva, WI, United States.

[B93] Samejima F. (1969). Estimation of latent ability using a response pattern of graded scores. Psychometrika 34, 1–97. 10.1007/BF03372160

[B94] Sannert R. Krell M. (2023). A professional development program to foster science teachers' professional competence, enhance classroom practice and improve student outcomes related to scientific reasoning. Prog. Sci. Educ. 6, 47–62.

[B95] Schunk D. H. Meece J. L. (2006). “Self-efficacy development in adolescence” in Self-efficacy Beliefs of Adolescents, eds F. Pajares, and T. C. Urdan (Greenwich, CT: Information Age Publishing), 71–96.

[B96] Schwarz G. (1978). Estimating the dimension of a model. Ann. Stat. 6, 461–464. 10.1214/aos/1176344136

[B97] Settlage J. Southerland S. A. Smith L. K. Ceglie R. (2009). Constructing a doubt-free teaching self: self-efficacy, teacher identity, and science instruction within diverse settings. J. Res. Sci. Teach. 46, 102–125. 10.1002/tea.20268

[B98] Shirazi S. (2017). Student experience of school science. Int. J. Sci. Educ. 39, 1891–1912. 10.1080/09500693.2017.1356943

[B99] Shroyer G. Riggs I. M. Enochs L. (2014). “Measurement of science teachers' efficacy beliefs: the role of the science teaching efficacy belief instrument” in The Role of Science Teachers' Beliefs in International Classrooms: From Teacher Actions to Student Learning, eds R. Evans, J. Luft, C. Czerniak, and C. Pea (Rotterdam: SensePublishers), 103–118. 10.1007/978-94-6209-557-1_7

[B100] Sia A. P. (1992). “Pre-service elementary teachers' perceived efficacy in teaching environmental education: a preliminary study,” in Paper presentation at the Annual Meeting of the North American Association for Environmental Education, Toronto, Canada.

[B101] Smith E. V. Wakely M. B. De Kruif R. E. L. (2003). Optimizing rating scales for self-efficacy (and other) research. Educ. Psychol. Meas. 63, 369–391. 10.1177/0013164403063003002

[B102] Smolleck L. D. Zembal-Saul C. Yoder E. P. (2006). The development and validation of an instrument to measure preservice teachers' self-efficacy in regard to the teaching of science as inquiry. J. Sci. Teach. Educ. 17, 137–163. 10.1007/s10972-006-9015-6

[B103] Stöber J. (2001). The Social Desirability Scale-17 (SDS-17): convergent validity, discriminant validity, and relationship with age. Eur. J. Psychol. Assess. 17, 222–232. 10.1027//1015-5759.17.3.222

[B104] Talsma K. Schüz B. Schwarzer R. Norris K. (2018). I believe, therefore I achieve (and vice versa): a meta-analytic cross-lagged panel analysis of self-efficacy and academic performance. Learn. Individ. Differ. 61, 136–150. 10.1016/j.lindif.2017.11.015

[B105] Terhart E. (2019). “Teacher Education in Germany” in Oxford Research Encyclopedia of Education. Available online at: https://oxfordre.com/education/view/10.1093/acrefore/9780190264093.001.0001/acrefore-9780190264093-e-377 (accessed November 14, 2023).

[B106] Valentine J. C. DuBois D. L. Cooper H. (2004). The relation between self-beliefs and academic achievement: a meta-analytic review. Educ. Psychol. 39, 111–133. 10.1207/s15326985ep3902_3

[B107] van den Wollenberg A. L. (1982). Two new test statistics for the Rasch model. Psychometrika 47, 123–139. 10.1007/BF02296270

[B108] van der Linden W. J. (2016). “Unidimensional logistic response models” in Handbook of Item Response Theory: Three Volume Set, ed. W. J. van der Linden (New York, NY: Chapman and Hall/CRC), 13–30. 10.1201/9781315374512

[B109] van der Linden W. J. Hambleton R. K. (1997). Handbook of Modern Item Response Theory. New York, NY: Springer New York. 10.1007/978-1-4757-2691-6

[B110] Van Dusen B. Nissen J. (2019). “Criteria for collapsing rating scale responses: a case study of the CLASS,” in Paper presentation at the Physics Education Research Conference 2019, Provo, UT, United States. 10.1119/perc.2019.pr.Van_Dusen

[B111] Vandekerckhove J. Matzke D. Wagenmakers E.-J. (2015). “Model comparison and the principle of parsimony” in The Oxford Handbook of Computational and Mathematical Psychology, eds Busemeyer J. R. Wang Z. Townsend J. T. Eidels A. (Oxford: Oxford University Press), 300–319. 10.1093/oxfordhb/9780199957996.013.14

[B112] Velthuis C. Fisser P. Pieters J. (2014). Teacher training and pre-service primary teachers' self-efficacy for science teaching. J. Sci. Teach. Educ. 25, 445–464. 10.1007/s10972-013-9363-y

[B113] von Davier M. (2016). “Rasch model” in Handbook of Item Response Theory: Three Volume Set, ed. W. J. van der Linden (New York, NY: Chapman and Hall/CRC), 31–48.

[B114] Wechsler D. (2008). Wechsler Adult Intelligence Scale – 4th Edition (WAIS-IV). San Antonio, TX: Pearson Assessment. 10.1037/t15169-000

[B115] Welter V. D. E. Herzog S. Harms U. Steffensky M. Großschedl J. (2022). School subjects' synergy and teacher knowledge: do biology and chemistry teachers benefit equally from their second subject? J. Res. Sci. Teach. 59, 285–326. 10.1002/tea.21728

[B116] Wilson E. B. Hilferty M. M. (1931). The distribution of chi-square. *Proc. Natl. Acad. Sci*. U.S.A. 17, 684–688. 10.1073/pnas.17.12.684 16577411 PMC1076144

[B117] Wright B. D. Linacre J. M. (1994). Reasonable mean-square fit values. Rasch Meas. Trans. 8, 370.

[B118] Wright B. D. Masters G. N. (1982). Rating Scale Analysis. Chicago, IL: Mesa Press.

[B119] Wu M. Adams R. (2007). Applying the Rasch Model to Psycho-social Measurement: A Practical Approach. Melbourne, VIC: Educational Measurement Solutions.

[B120] Zander L. Höhne E. Harms S. Pfost M. Hornsey M. J. (2020). When grades are high but self-efficacy is low: unpacking the confidence gap between girls and boys in mathematics. Front. Psychol. 11, 552355. 10.3389/fpsyg.2020.552355 33162905 PMC7580255

[B121] Zee M. Koomen M. Y. (2016). Teacher self-efficacy and its effects on classroom processes, student academic adjustment, and teacher well-being: a synthesis of 40 years of research. Rev. Educ. Res. 86, 981–1015. 10.3102/0034654315626801

