# Supplementary material for: Adapting a self-efficacy scale to the task of teaching scientific reasoning: collecting evidence for its psychometric quality using Rasch measurement
Source: Front Psychol. 2024 Feb 7;15:1339615. doi: 10.3389/fpsyg.2024.1339615 (PMC10879573; doi:10.3389/fpsyg.2024.1339615)
Supplement: Supplementary file 1 [file Data_Sheet_1.DOCX]

Supplementary Material

Adapting a Self-Efficacy Scale to the Task of Teaching Scientific Reasoning: Collecting Evidence for its Psychometric Quality Using Rasch Measurement

Virginia Deborah Elaine Welter, Merryn Dawborn-Gundlach, Leroy Großmann, Moritz Krell*

*** Correspondence:** Prof. Dr. Moritz Krell: [krell@leibniz-ipn.de](mailto:krell@leibniz-ipn.de)

# Supplementary Information on the Wording of the TSR-EBI Items

## German TSR-EBI Items (Based on STEBI; Riggs and Enochs, 1990)

1. Ich werde immer besser darin, wissenschaftsmethodische Kompetenzen im Biologieunterricht zu fördern.
2. Auch wenn ich mich sehr anstrenge, fördere ich wissenschaftsmethodische Kompetenzen weniger gut als andere Bereiche des Biologieunterrichts.
3. Es fällt mir leicht, Biologiestunden zu planen, in denen wissenschaftsmethodische Kompetenzen meiner Schüler*innen gefördert werden.
4. Ich bin nicht sehr gut darin, Schüler*innen bei der Durchführung von Methoden zur naturwissenschaftlichen Erkenntnisgewinnung zu begleiten.
5. Ich fördere wissenschaftsmethodische Kompetenzen grundsätzlich nicht sehr erfolgreich bei meinen Schüler*innen.
6. Ich verstehe die Methoden zur naturwissenschaftlichen Erkenntnisgewinnung selbst gut genug, um diese im Biologieunterricht erfolgreich zu unterrichten.
7. Ich finde es schwierig, meinen Schülern*innen Methoden zur naturwissenschaftlichen Erkenntnisgewinnung zu erklären.
8. Ich bin üblicherweise in der Lage, die Fragen meiner Schüler*innen über Methoden zur naturwissenschaftlichen Erkenntnisgewinnung zu beantworten.
9. Ich frage mich, ob ich die notwendigen Fähigkeiten habe, wissenschaftsmethodische Kompetenzen zu fördern.
10. Wenn ich die Wahl hätte, würde ich die Schulleitung bitten, in meinen Stunden zur Förderung wissenschaftsmethodischer Kompetenzen nicht zu hospitieren.
11. Wenn Schüler*innen Schwierigkeiten haben, eine bestimmte Methode zur naturwissenschaftlichen Erkenntnisgewinnung zu verstehen, bin ich normalerweise ratlos, wie ich ihnen helfen kann, diese besser zu verstehen.
12. Wenn ich wissenschaftsmethodische Kompetenzen fördern möchte, freue ich mich in der Regel über Fragen der Schüler*innen.
13. Ich weiß nicht, was ich tun soll, um Schüler*innen für die Methoden zur naturwissenschaftlichen Erkenntnisgewinnung zu begeistern.

## English Translation of the German TSR-EBI Items (Based on STEBI; Riggs and Enochs, 1990)

1. I am continually finding better ways to teach scientific reasoning in my biology class.
2. Even when I try very hard, I don’t teach scientific reasoning as well as I do most other topics in my biology class.
3. I know the steps necessary to teach scientific reasoning effectively in my biology class.
4. I am not very effective in monitoring students’ scientific reasoning.
5. I generally teach scientific reasoning ineffectively.
6. I understand concepts of scientific reasoning well enough to be effective in teaching them in my biology class.
7. I find it difficult to explain scientific reasoning to students.
8. I am typically able to answer students’ questions about scientific reasoning.
9. I wonder if I have the necessary skills to teach scientific reasoning.
10. Given a choice, I would not invite the principal to evaluate my teaching on scientific reasoning.
11. When a student has difficulty understanding a scientific reasoning concept, I am usually at a loss as to how to help the student understand it better.
12. When teaching scientific reasoning, I usually welcome student questions.
13. I don’t know what to do to turn students on to scientific reasoning.

# Supplementary Figures

**
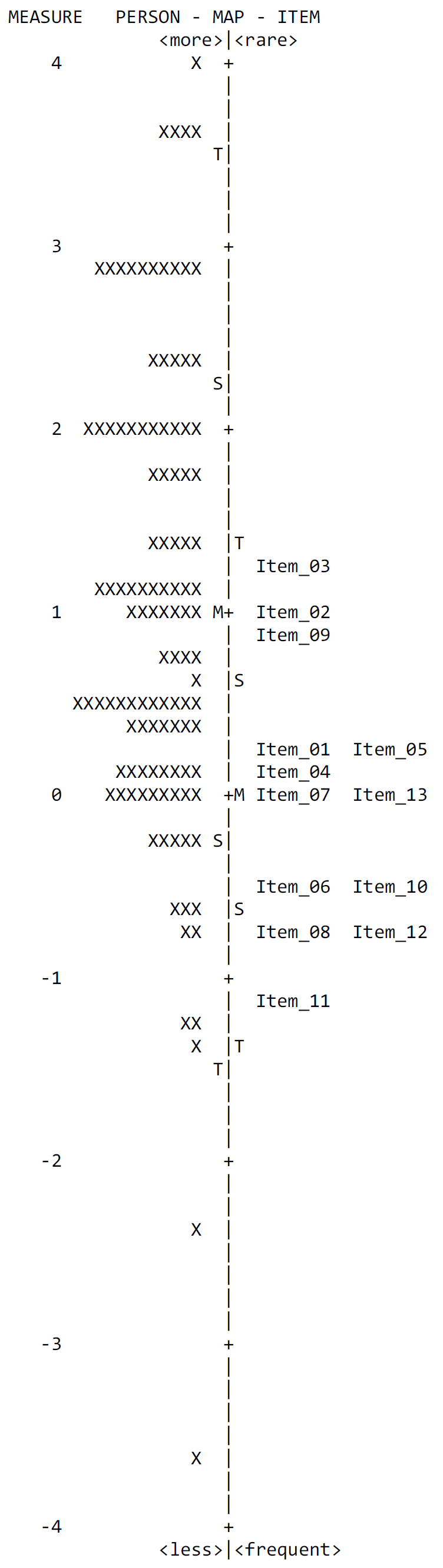
**

**Supplementary Figure 1.** Wright Map of the 3-Category TSR-EBI in the First Round of Validation.

**
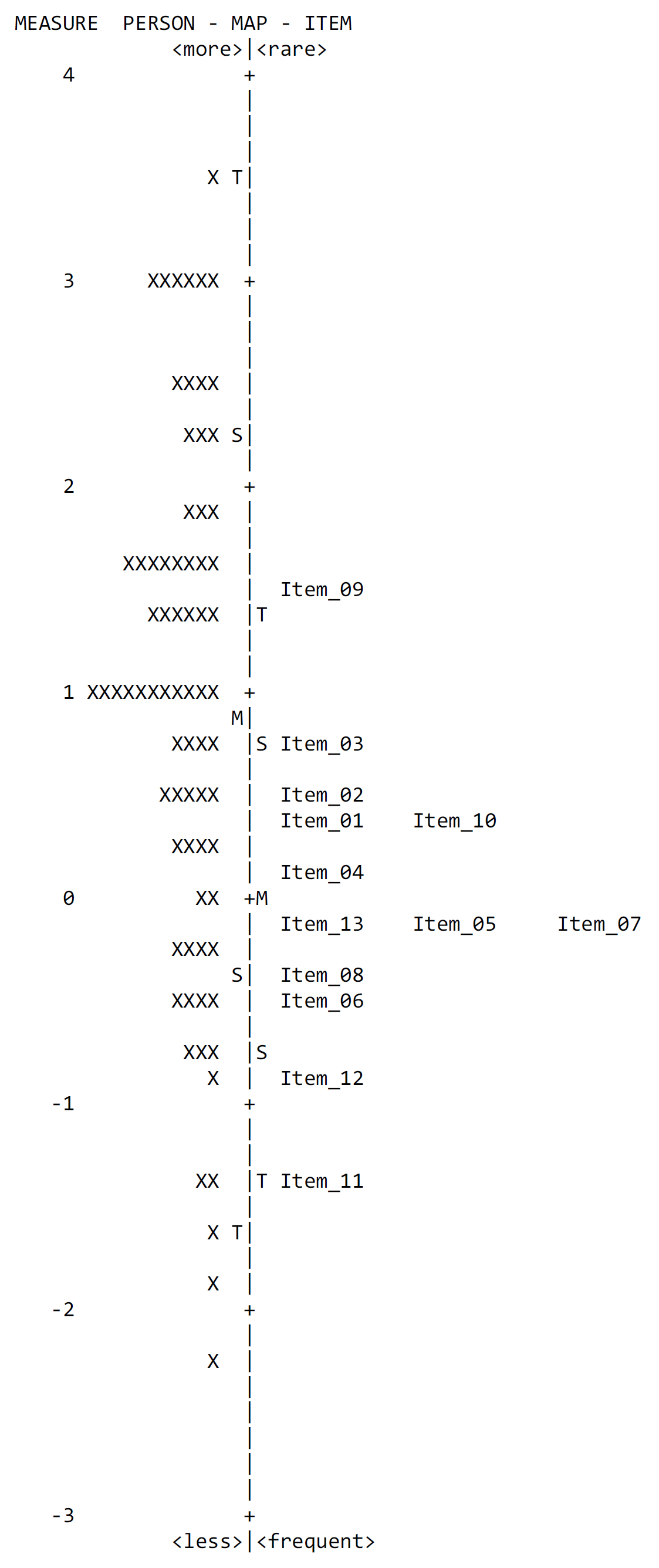
**

**Supplementary Figure 2.** Wright Map of the 3-Category TSR-EBI in the Cross-Validation.
